# Clutter suppression in ultrasound: performance evaluation and review of low-rank and sparse matrix decomposition methods

**DOI:** 10.1186/s12938-020-00778-z

**Published:** 2020-05-28

**Authors:** Naiyuan Zhang, Md Ashikuzzaman, Hassan Rivaz

**Affiliations:** Department of Electrical and Computer Engineering, Concordia, Rue Sainte-Catherine O, Montreal, Canada

**Keywords:** Ultrasound color flow imaging, Clutter suppression, Vessel visualization, Low-rank and sparse matrix decomposition

## Abstract

Vessel diseases are often accompanied by abnormalities related to vascular shape and size. Therefore, a clear visualization of vasculature is of high clinical significance. Ultrasound color flow imaging (CFI) is one of the prominent techniques for flow visualization. However, clutter signals originating from slow-moving tissue are one of the main obstacles to obtain a clear view of the vascular network. Enhancement of the vasculature by suppressing the clutters is a significant and irreplaceable step for many applications of ultrasound CFI. Currently, this task is often performed by singular value decomposition (SVD) of the data matrix. This approach exhibits two well-known limitations. First, the performance of SVD is sensitive to the proper manual selection of the ranks corresponding to clutter and blood subspaces. Second, SVD is prone to failure in the presence of large random noise in the dataset. A potential solution to these issues is using decomposition into low-rank and sparse matrices (DLSM) framework. SVD is one of the algorithms for solving the minimization problem under the DLSM framework. Many other algorithms under DLSM avoid full SVD and use approximated SVD or SVD-free ideas which may have better performance with higher robustness and less computing time. In practice, these models separate blood from clutter based on the assumption that steady clutter represents a low-rank structure and that the moving blood component is sparse. In this paper, we present a comprehensive review of ultrasound clutter suppression techniques and exploit the feasibility of low-rank and sparse decomposition schemes in ultrasound clutter suppression. We conduct this review study by adapting 106 DLSM algorithms and validating them against simulation, phantom, and in vivo rat datasets. Two conventional quality metrics, signal-to-noise ratio (SNR) and contrast-to-noise ratio (CNR), are used for performance evaluation. In addition, computation times required by different algorithms for generating clutter suppressed images are reported. Our extensive analysis shows that the DLSM framework can be successfully applied to ultrasound clutter suppression.

## Background

Angiology, which concerns vessel-related diseases, is one of the most important branches of medical science since vascular diseases are very common and cause death to a large number of people every year [1]. Vascular diseases can primarily be divided into several categories based on the type of vessel. Arterial diseases include aneurysms, thrombosis, vasculitides, and vasospastic disorders. Venous diseases include venous thrombosis, chronic venous insufficiency, and varicose veins. There are also diseases associated with capillaries. One such example is the capillary hemangioma. Currently, the most accepted classification of vascular abnormalities is tumors and deformities which were adopted in 1996 by the International Society for the Study of Vascular Anomalies [2]. Therefore, many major clinical diseases have been shown to cause vascular growth abnormalities. For example, many cardiovascular diseases are related to aneurysms or other vascular variations [3, 4]; the growth of many tumors in cancer is also highly dependent on angiogenesis [5, 6]. Similarly, angiogenesis is also an important feature of diabetes-related diseases [7–9] and endometriosis [10]. Therefore, blood vessel imaging is indispensable in clinical fields and medical research [11], including but not limited to diagnosis, treatment planning, surgery, and follow-up treatment results.

Several medical imaging modalities such as duplex ultrasound (DUS), computed tomography (CT), magnetic resonance imaging (MRI), and digital subtraction angiography (DSA) have been employed thus far to ensure a proper visualization of blood vessels. Among different vascular imaging modalities, ultrasound has become the primary choice, for it is safe, economical, easy-to-use, and most importantly, real-time [11]. Duplex ultrasound is the combination of color flow imaging (CFI) and grayscale/brightness mode (B-mode) imaging, whereas the CFI is used to observe the blood flow direction and velocities, and the B-mode ultrasound is used to visualize two-dimensional anatomy images simultaneously. By simultaneous processing frequency, phase, and amplitude of the backscattered ultrasound signal, CFI can rapidly identify the flow direction and velocities in the region of interest. Moreover, CFI can be used to mark flow abnormalities, including stenoses and occlusions [12]. The comparison between ultrasound and other vascular imaging methods is shown in Table 1.

**Table 1:** A comparison of vessel imaging methods [11, 13]

	Acquisition time (min)	Safety	Limitations
MRI	30	No risk	Long imaging time. No vessel wall. Metal Prohibited
CT	5	Low risk	Radiation risk. Complication risk
DSA	120	Low risk	Radiation risk. Complication risk. Invasive
DUS	15	No risk	Limit resolution. Prohibited at wound sites
			High-level user-dependent. Obstruction of gas and solid

Acquisition time is approximate with pretreatments and acquisition included

**Fig. 1:**
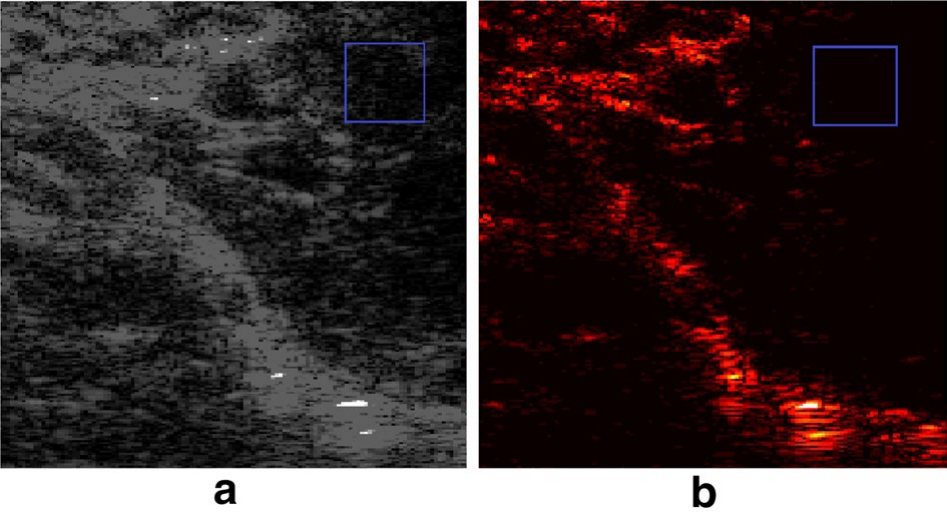
A set of comparison images showing CFI with and without clutter filters. **a** CFI raw data in Brightness mode. **b** The same data after clutter suppression by SVD. In the upper right window, the raw CFI data contain a lot of tissue clutter in the background, which is suppressed by SVD in the second image

Due to the excellent performance, ultrasound CFI has been increasingly used for the diagnosis of vessel-related diseases [14]. However, as one of the most promising and widely applicable methods with low cost and no risk, CFI still has some obvious disadvantages. First, due to the tissue scattering of the ultrasound beam, the intensity of the blood backscatter is several orders of magnitude less than that of the tissue backscatter, which makes it hard to image blood flow clearly [12, 15]. Second, more than three pulses are needed to estimate the velocity because of the stochastic behavior of blood signals and the impact of tissue clutters [12]. The requirement for multiple pulses limits frame rates and the number of scan lines. Third, CFI is limited by the insonation angle which is the angle between the ultrasound beam and the flow direction [16, 17]. Generally, an accurate measurement requires Doppler angles ranging from 30° to 60°, where smaller angles will result in lower speeds and greater angles will produce a significant overestimation of the velocity [16, 17]. Last but not least, blood signals and clutter signals will possess a significant overlap, that is, when the blood flow rate is very slow (such as in small blood vessels) or when the tissue movement is obvious. The overlap will be harmful to blood vessel visualization [18, 19]. Most of these disadvantages are caused by clutter, as a consequence, clutter suppression is particularly important in ultrasound blood flow imaging. Figure 1 shows the clutter in two CFI images and illustrates the importance of clutter filtering.

The main purpose of clutter filtering is to suppress gross-moving tissue clutter and beam sidelobe leakages [19]. An efficient clutter suppression is a prerequisite for CFI to present accurate and clear blood flow maps. The most significant effect of clutter reduction is an increase in the signal-to-noise ratio (SNR) of the blood signal, which enables clearer blood flow maps and reduces erroneous moving tissue signals. Meanwhile, pure blood flow signals also help reduce the number of pulses needed to estimate the speed, thereby increasing the frame rate. In addition, the overlapping frequency spectra of slow blood flow and fast-moving tissue will no longer hinder the microvascular flow detection or add bias to high-velocity flow [19, 20].

However, the perfect removal of clutter signals is still impossible for now since clutter signals are 40 to 100 dB stronger than blood signals and they exhibit similar properties [15].

In early development of CFI clutter filtering, tissue signals and blood signals were assumed to have completely different frequency characteristics. This assumption holds that tissue and blood signals exhibit non-overlapping frequency spectra since the tissue is considered to be nearly stationary whereas red blood cells are rapidly moving [18]. Based on this assumption, finite impulse response (FIR) and infinite impulse response (IIR) high-pass filters were used to filter clutter signals and enhance the sensitivity of blood flow [15, 18]. Nowadays, it is recognized that FIR and IIR filters have distinct drawbacks. FIR filters require a high order to separate blood from clutter, whereas IIR filters take a long time to settle [18, 21]. Furthermore, both types of high-pass filters suffer from the insufficient number of slow time samples, which leads to inefficient suppression of clutter [19, 22]. Another clutter removal approach introduces linear regression filters [23–25]. The regression filter eliminates clutter signals by taking the least square fitting of signals from the signal model [18]. Studies suggest that polynomial regression filters and IIR filters have better performance than FIR filters. In the case of contrast-enhanced ultrasound vascular imaging, pulse inversion technique has been introduced toward the end of clutter rejection [26–28]. In this approach, the linearity property of tissue echo is exploited for distinguishing tissue from blood [26, 29, 30]. Although these methods significantly improve the SNR of blood signals, they are not considered in this paper because of their invasiveness.

The aforementioned traditional clutter suppression algorithms, such as FIR and IIR, have at least one of the following issues: (1) long settling time, (2) inability to adaptively suppress the clutter based on data property, (3) inadequate temporal sample or resolution. Besides, two main reasons are resulting in the imperative innovation of ultrasound clutter filtering. First, new ultrasound technologies like plane wave ultrasound have brought a higher frame rate and imaging speed. Traditional filters cannot meet the higher clutter filtering performance requirements, though they do not suffer from the settling time due to the high frame rate. Second, the underlying assumption of traditional filters does not hold in the presence of significant tissue motion stemming from the sonographer’s sinusoidal hand movement or the patient’s breathing and heartbeat [31, 32]. In such a scenario, the frequency bands corresponding to tissue and blood overlap with each other without a definite boundary between them. Hence, high-pass filters fail to separate blood from tissue when the clutter signal dominates with non-zero Doppler frequency caused by substantial tissue movements.

To resolve these issues, eigen-based filters [33–35] have been proposed which take both spatial and temporal samples into consideration to develop an adaptive clutter suppression scheme. The techniques related to these eigen-based filters have been widely applied in the field of computer science which is mainly used for processing high-dimensional data. Meanwhile, these techniques are not based on incomplete traditional assumptions. Matrix decomposition is the principal idea behind these algorithms and it is assumed that clutter and blood signals lie in different subspaces. Therefore, eigen-based filters are considered adaptive to gross motions induced by the sonographer or the subject being examined. Based on different assumptions, research proves that eigen-based filters perform better than traditional methods [20, 22].

Most of the eigen-based filters for ultrasound clutter suppression are based on singular value decomposition (SVD) or eigenvalue decomposition and improve upon it [36–39]. To perform the subspace separation task, slow-time temporal ultrasound frames are stacked as columns of data matrix, known as the Casorati matrix [40]. The SVD of this Casorati matrix provides the opportunity to distinguish blood from clutter. It has been reported in the literature that the most dominant singular values and vectors correspond to clutter, the next few represent blood and the least significant ones correspond to noise [41]. In these eigen-based approaches, the eigen or singular values representing clutter and noise are set to zero to find the blood component of the echo signal [41, 42].

Many SVD-based techniques have been proposed which work with conventional line-by-line scanning [20, 43–45]. These methods suffer from lacking an adequate number of temporal samples due to low frame rate associated with focused ultrasound imaging [21]. Recent clutter suppression algorithms [18, 42, 46–49] have resolved this issue by incorporating ultrafast plane-wave imaging. However, the blood signal in plane-wave ultrasound is even weaker than normal ultrasound due to the unfocused wave [50, 51]. The sidelobe in plane-wave imaging is also much higher than that in conventional imaging due to the same reason. Therefore, plane-wave ultrasound has a higher and more urgent filtering requirement than traditional CFI. Recent methods have extended SVD-based clutter suppression to a higher order by analyzing a data tensor instead of a two-dimensional matrix [42, 48, 52]. Since the first few singular values do not necessarily correspond to the clutter signal in the presence of a large temporal misalignment among the frames, the motion correction step has been introduced in SVD-based clutter rejection [53]. Since SVD was initially combined with plane-wave imaging in 2015, almost all the clutter suppression research have been based on plane-wave ultrasound since SVD can reach its full potential on large datasets [18].

**Fig. 2:**
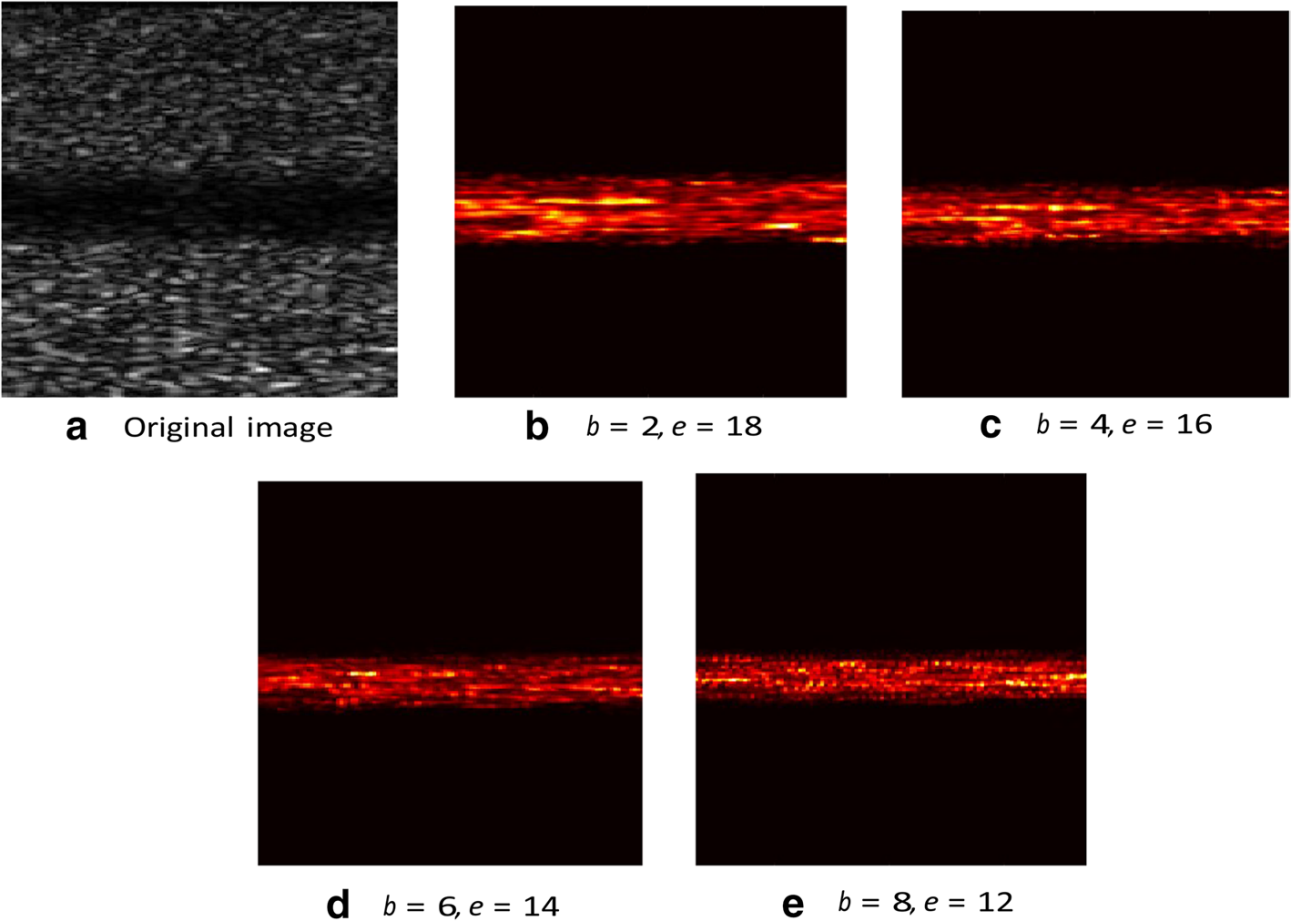
A set of pictures showing the threshold selection of SVD. **a** The original simulation data in brightness mode. **b**–**e** The processed images by SVD with different thresholds. Parameters *b* and *e* represent the selected rank of blood and noise signal, respectively. The full rank of the data is 20

Although SVD-based techniques are promising for suppressing clutter optimally, they have two major drawbacks. First, there is still no uniform and efficient standard for rank selection which presents boundaries of tissue and blood flow [42]. Proper subspace rank selection, which is done by extensive manual intervention, is crucial for the optimality of clutter rejection. Recent methods suggest different criteria for selecting the optimal ranks [54]. In addition, [21] proposes K-means clustering of the decomposed components as a criterion for selecting singular values and vectors corresponding to clutter and blood. Though different ideas are proposed for automatic rank selection [55], there is still no efficient and standard method. An example that briefly explains the problem of SVD threshold selection is shown in Fig. 2. The selected rank will affect blood signals. A large threshold range cannot effectively filter clutter and noise, and a small range will lose part of the blood signals. The second drawback is that SVD is sensitive to noise. It fails to obtain the optimal result while processing data with large random noise [56].

The aforementioned issues can potentially be resolved by taking the framework called decomposition into low-rank and sparse matrices (DLSM) [21] into account. SVD is one of the calculation methods in DLSM framework and there are also approximate SVD or SVD-free algorithms. This is a well-established framework in the field of computer vision due to its robustness to large noise and information corruption [56]. The underlying assumption of this approach is that steady tissue is a low-rank component and moving blood exhibits sparsity [50]. It has been noticed that both temporal and spatial information can be used to separate tissue and blood signals since tissue signals have a higher temporal–spatial coherence than blood signals (e.g., the blood scatterers are unique and constantly changing). A convex optimization problem is usually solved to decompose the data matrix into low-rank clutter and sparse blood components. A recent technique has used this model for the concurrent removal of clutter and noise [57]. Furthermore, recent work has incorporated deep learning with low-rank and sparse decomposition for improved clutter suppression performance [21].

The main purpose of this work is to demonstrate the feasibility of 106 established low-rank and sparse decomposition algorithms in ultrasound clutter suppression and to provide suggestions for most suitable DLSM models, optimization methods, and algorithms for ultrasound clutter suppression. The paper is organized as follows. “Decomposition into low-rank and sparse matrices (DLSM) framework” section illustrates DLSM framework including decomposition types, loss functions, and relationships with tensor decomposition. “Experiments” section includes detailed experimental settings and results on simulation, phantom, and in vivo rat datasets. Finally, the paper reveals our discoveries in “Discussion” and “Conclusion” sections including the appropriate DLSM algorithms for clutter suppression and the shortcomings of the remaining algorithms. Comments and possible solutions are also proposed in response to different shortcomings.

## Decomposition into low-rank and sparse matrices (DLSM) framework

Low-rank and sparse structures are attractive since they usually represent part of the large and high-dimensional data which we are most interested in. Noise and data corruption can be fixed when decomposing matrices into low-rank and sparse components. Methods like sparse representation and low-rank modeling have achieved great success in computer vision, natural language processing, system identification, bioinformatics, etc. [58–60]. So far, many different models, optimization methods, and algorithms are proposed aiming at solving the low-rank and sparse matrix recovery problems. Meanwhile, many classifications have been proposed [58, 61–63] according to linearity, convexity, number of subspaces, or number of addition matrices.

Decomposition into low-rank and sparse matrices (DLSM) is one of the relatively detailed and comprehensive frameworks [61] which classifies various models of matrix decomposition according to the number of constrained component matrices. DLSM framework provides a suitable framework for signal processing, system identification, computer vision, machine learning, etc. This decomposition idea is becoming more popular and widely used in recent years, especially after the robust principal component pursuit (RPCP) was purposed in papers of Candes et al. [56], and Chandrasekharan et al. in 2009 [64]. In the beginning, these algorithms are designed to deal with high-dimensional data which are often regarded as an extremely high-dimensional data matrix. Since many dimensions are usually independent, it is possible to recover the matrix from corruption or noise. These ideas are based on the assumption that the uncorrupted information matrix is highly correlated within the observing time-window and therefore lies in the low-rank subspace. At the same time, the moving foreground objects, noise, or other special signals constitute the correlated sparse outliers.

Based on similar assumptions, several algorithms under DLSM framework have been validated that they can be successfully applied to ultrasound clutter suppression [18, 22, 36, 38, 55, 65]. In medical ultrasound, tissue and blood flow also lie in different subspace. In terms of temporal information, tissue signals and blood signals have different spectral features due to the different movement patterns of blood and tissue. As for spatial features, the blood signal has an extremely lower spatial coherence than tissue signal because the irregular movement and arrangement of red blood cells produce constantly changing scatterers, whereas the tissue movement is overall patterned. Therefore, they gain a low rank and sparsity characteristics, respectively, and lie in different subspaces. Due to the robust and efficient performance of DLSM frameworks in separating low-rank and sparse components, it can show great potential in the field of ultrasound clutter suppression.

**Fig. 3:**
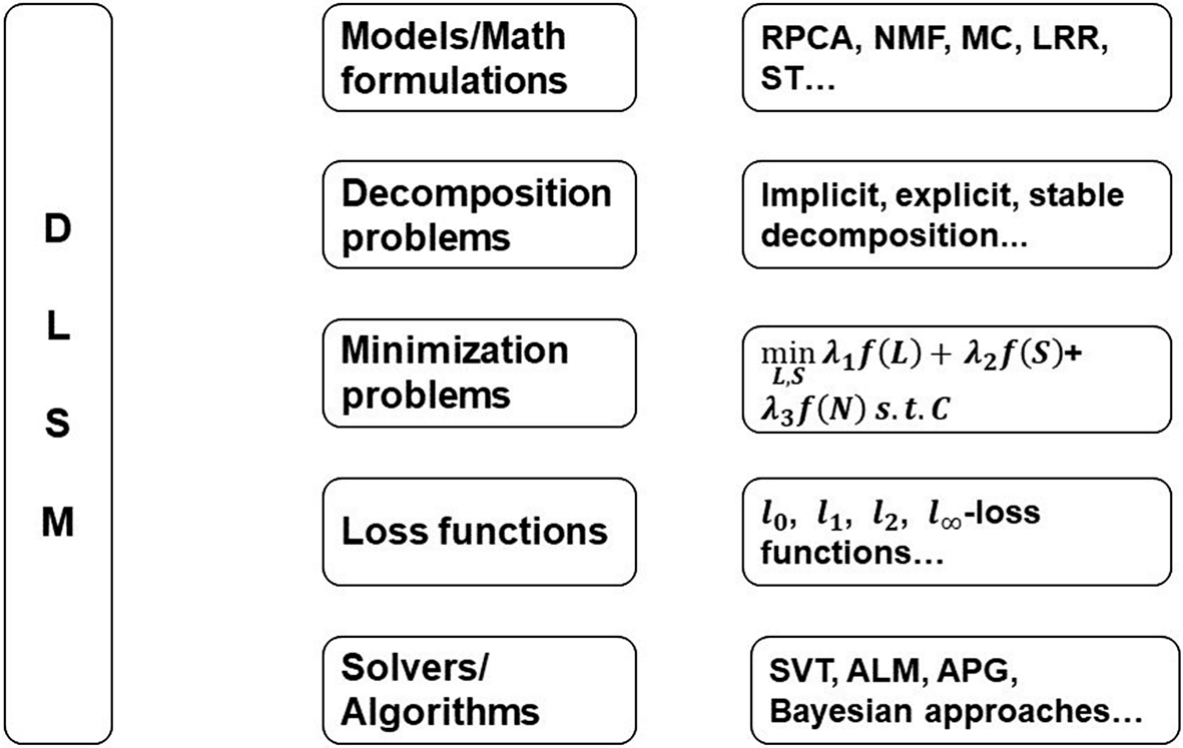
The schematic diagram of DLSM framework. DLSM framework contains 5 branches, which are models (or called math formulations), decomposition problems, minimization problems, loss functions, solvers (or called algorithms). Examples are shown beside the branches

Overall, DLSM framework is divided into decomposition problems, minimization problems, loss function and solvers (algorithms used to solve the optimization problems) [63] as Fig. 3 shows. The permutations and combinations of models and optimization methods and solvers lead to various algorithms, which is the origin of the DLSM framework. DLSM framework and its application in the ultrasound clutter suppression will be briefly illustrated in the following subsections.

### Preprocessing and notations

Preprocessing of ultrasound data is necessary for integration into an input matrix or tensor in a special shape when applying DLSM algorithms. In general, the input of the DLSM algorithm consists of a sequence of *n* consecutive ultrasound data ($F_1\dots F_n$) with the original size of $F\in \mathfrak {R}^{i_1\times i_2}$. For a two-dimensional DLSM algorithm, the input *M* ($M\in \mathfrak {R}^{m\times n}, m=i_1\times i_2$) is in matrix form in most cases which consists of *n* resized ultrasound data frames ($F\in \mathfrak {R}^{m\times 1}$) arranged in order. In terms of high-order DLSM algorithms, the input is typically an *N*-order tensor *T* ($T\in \mathfrak {R}^{t_1\times t_2\dots t_n}$). *T* is generally third order and concatenated by original size ultrasound frames, where $T=[F_1^{i_1\times i_2},\dots F_n^{i_1\times i_2}], T\in \mathfrak {R}^{i_1\times i_2\times n}$. Next, the input *M* (or *T*) is decomposed into several components through the DLSM algorithm as follows:

1 $$\begin{aligned} M = \sum \limits _{x=1}^X K_x \end{aligned}$$

where $0\le X \le 3$ and $K_1,K_2,K_3$ typically represent low-rank *L*, sparse *S*, and noise components *E*, respectively. The specific components $K_x$ and the number of *X* depend on the purpose (interested in sparse or low rank components) and the decomposition formulation.

### Decomposition formulations

#### Implicit decomposition

Implicit decomposition $(X=1)$: Under the condition that *x* is equal to 1, matrix *M* is approximately equal to a target low-rank matrix *L* under the constraint condition, because the information that people interested in mainly lies in the low rank component in most cases. Sparse matrix *S* can be obtained from the difference between *M* and *S* (e.g., $S=M-L$). However, this processing is the opposite in the application of ultrasound clutter suppression because the blood signal is relatively sparse. The formulation of this problem is as follows:

2 $$\begin{aligned} \min \, f(M,L) \quad \text {s.t.}\; L \end{aligned}$$

where $M\approx L$, *f*(.) is a loss function used for the minimization term which depends on specific solvers or algorithms. Models like principal component analysis (PCA), non-negative matrix factorization (NMF), and matrix completion (MC) are in this category.

For the applications targeted to sparse components, implicit decomposition sets the target matrix $K_1$ as a sparse matrix *S*. Then low-rank matrix *L* is the difference between *M* and *S* which can be calculated as $L=M-S$. Sparse dictionary learning [66], sparse linear approximation, and compressive sensing [66–68], etc. are built under the same idea.

3 $$\begin{aligned} \min \, f(M,S) \quad \text {s.t.}\; S \end{aligned}$$

where $M\approx S$, and the difference contains noise and other information. In this case, implicit decomposition can be used in the compressed sensing and signal recovery similar to unsupervised clustering [69] and image recognition [70].

Before more robust explicit decomposition method was proposed, the main development of ultrasound clutter suppression was based on PCA or SVD or eigenvalues, which belong to implicit decomposition [19, 22, 35, 38, 39, 45]. Although many experiments have proved that these eigen-based filters greatly improve the performance than traditional IIR and regression filters, many authors realize that the filtering method based on implicit decomposition is not robust to accelerated tissue movements and different kinds of noise [22, 35, 39]. Moreover, their expensive computational complexity is not suitable for real-time processing.

#### Explicit decomposition

Explicit decomposition $(X=2)$: Under this condition, *M* is usually decomposed into a low-rank matrix $K_1=L$ and a sparse matrix $K_2=S$ ($M\approx L+S$). This is called explicit decomposition because there are two constraints. One is sparse constraint over *S* and the other is low-rank constraint over *L*. Therefore, explicit decomposition is more robust than implicit decomposition. The formulation of explicit decomposition is as follows:

4 $$\begin{aligned} \min \, f(L)+f(S) \quad \text {s.t.}\; L,S \end{aligned}$$

where $M\approx L+S$ and *f*(.) represents loss function. The explicit decomposition includes robust principal component analysis (RPCA), robust non-negative matrix factorization (RNMF), robust matrix completion (RMC), and robust subspace tracking (RST) [59, 71].

These methods generally work better and are more robust than implicit decomposition because of the additional constraints [71]. In this way, RPCA has been used as a powerful tool in MRI, CT, and ultrasound imaging [72–74]. Many optimization algorithms have been proposed for cluster suppression in ultrasound imaging using RPCA, RMC [21, 55, 75].

#### Stable decomposition

Stable decomposition $(X=3)$: Due to the fact that there are always noise and corruption caused by special cases in the real world, an additional matrix $K_3$ is added to represent unexpected components. $K_3$ could represent distortion, shadows, and noise according to special situations ($M\approx S+L+N$). It is more stable than the explicit decomposition since more detailed information is separated and taken into account. The stable decomposition can handle more complex situations in the real life such as dynamic videos and maritime monitoring videos which are corrupted by complicated noise.

5 $$\begin{aligned} \min \, f(S)+f(L)+f(N) \quad \text {s.t.}\; L, S \end{aligned}$$

Stable decomposition methods include Stable Principal Component Analysis (Stable PCA) or Stable Non-negative Matrix Factorization (Stable NMF) and Three Term Decomposition (TTD). These methods can deal with more complex situations. In terms of US imaging, it is usually assumed that signal *M* contains clutter signals *L* (low-rank), blood signals *S* (sparse), and noise *N*. Since ultrasound signals have complex noises and dynamic clutter signals, this assumption $M=S+L+N$ is more acceptable when there are meticulous requirements such as microvascular imaging. Although some literature mentions the stable decomposition of blood (*L*, *S*, *E* respectively represent blood flow signals, clutter signals, and noise), they do not illustrate whether constraints are added to noise component. Therefore, the stable decomposition formulation is still a promising research area for ultrasound clutter suppression.

### Models under DLSM framework

As of today, many models, also called problem formulations, have been proposed. According to different math formulations and features, methods are usually classified under families such as robust principal component analysis (RPCA), non-negative matrix factorization (NMF), matrix completion (MC), and Subspace Tracking (ST). Different models have different functions and aims. However, it has been proved that the solutions of many robust models can be mutually expressed in closed forms [76]. For instance, RPCA via principal component pursuit [56] can be considered as MC models using $l_1$-norm loss function [63]. In addition, these models can be flexibly generated in any decomposition formulations. For example, adding constraints on noise components based on the RPCA will change it from explicit decomposition to stable decomposition.

#### Robust principal component analysis

Principal component analysis (PCA) generates a set of linearly uncorrelated variables which is called principal components, from a set of observations by orthogonal transformation. Similar mathematical tools include SVD and eigenvalue decomposition. RPCA is based on the extension of PCA (expansion from implicit decomposition to explicit decomposition), which aims to recover low-rank components and reduce the impact of grossly corrupted data. RPCA can be approached by principal component pursuit (PCP) [56, 64], Bayesian RPCA [77–79], and so on. RPCA problem is generally expressed as follows:

6 $$\begin{aligned} M = L + S \end{aligned}$$

where *L* is low-rank matrix and *S* is sparse matrix. According to the nature of *L* and *S*, the most intuitive way to solve the RPCA problem is to minimize the rank of *L* and the $l_0$-norm of *S*:

7 $$\begin{aligned} \min _{L,S}\, rank(L) + \lambda \Vert S\Vert _{l_0}\quad \text {s.t.}\;M-L-S=0 \end{aligned}$$

where $\lambda$ is a balanced parameter. However, this formulation is *NP*-hard. Therefore, optimization problems like PCP are needed.

The convex optimization principal component pursuit (PCP) was first proposed by Candes et al. [56, 63, 80] to address the RPCA problem. It becomes one of the most famous methods of face recognition and background modeling in recent years. PCP uses the following formula to convexly optimize RPCA problem:

8 $$\begin{aligned} \min _{L,S}\, \Vert L\Vert _{*} + \lambda \Vert S\Vert _{l_1}\quad \text {s.t.}\;M-L-S=0 \end{aligned}$$

where $\Vert .\Vert _{*}$ and $\Vert .\Vert _{l_1}$ are the nuclear norm and $l_1$-norm, respectively. Although this method excels in computer vision, there are still some limitations to sparse components recovery. First, it requires expensive computational algorithms. Second, it is a batch method which is not suitable for real-time applications, especially for plane-wave ultrasound with high frame rates. Third, it has very high requirements for low rank and sparse properties; however, the complex blood flow or noise may make it difficult for ultrasound data to meet such requirements. To accelerate the algorithms and achieve higher precision, different solvers have been developed [81–83]. Solvers for real-time implementations have also been proposed [84, 85].

The stable principal component pursuit (SPCP) is a stable expanded form based on PCP, which mainly aims at reducing the impact of noise. SPCP adds noise term *E* based on PCP and constrains it by Frobenius norm.

#### Matrix completion

The main purpose of matrix completion (MC) is to recover low-rank observation matrix of its missing entries. The Netflix movie rating matrix recover problem is one of the most classic examples. The classic low-rank matrix completion problem can be seen as finding the lowest rank of the matrix *L* which matches the matrix *M*, for all the measured entries in set $\Omega$. The mathematical formulation of MC problem is as follows:

9 $$\begin{aligned} \min _{L}\, rank(L)\quad \text {s.t.}\;L_{m,n}=M_{m,n}\;\forall i,j\in \Omega \end{aligned}$$

Due to the implicit decomposition of MC is not robust to noise which only affects a small-scale data [86, 87], MC is generally extended to explicit decomposition by adding restrictions, which is called robust matrix completion (RMC). The common RMC obtains stronger robustness than MC by adding sparse constraints, and its formulation after convex optimization is as follows:

10 $$\begin{aligned} \min _{L,S}\, \Vert L\Vert _{*} + \lambda \Vert S\Vert _{l_1}\quad \text {s.t.}\;P_\Omega (L+S)=P_\Omega (M) \end{aligned}$$

where $P_\Omega (M)$ is the projection of the complete dataset on the measured entries $\Omega$. Although the form of decomposition is the same as PCP, the unique constraints of RMC make it supervised while the PCP is unsupervised learning [63], which is consistent with the purpose of RMC.

#### Nonnegative matrix factorization

The nonnegative matrix factorization (NMF) is also a widely used matrix factorization and dimension reduction model under DLSM framework. The main unique feature of NMF is that low-rank factor matrix is subject to nonnegative constraints consistent with the physically natural features in many fields [88, 89]. The NMF problem is generally expressed as follows:

11 $$\begin{aligned} M \approx WH^\top \end{aligned}$$

where $W\in \mathfrak {R}^{m\times k}$ and $H\in \mathfrak {R}^{n\times k}$ are two nonnegative matrices, and $k<\min \{m,n\}$ due to the goal of dimension reduction. The most common formulation for the optimization problem of NMF is as following:

12 $$\begin{aligned} \min _{W,H}\, f(W,H) = \Vert M-WH^\top \Vert _F^2\quad \text {s.t.}\;W\ge 0,H\ge 0 \end{aligned}$$

where $\Vert .\Vert _F^2$ is the Frobenius norm. The problem (14) is a non-convex problem and it is *NP*-hard to find its global minimum [88, 90]. Consequently, optimization algorithms and solvers are developed for the local minimum.

#### Subspace tracking

The subspace tracking (ST) can be regarded as the dynamic RPCA designed to handle increasing new data or dynamic subspaces. The data at each moment *t* are processed as the increments and then discarded. This idea addresses the problem when new observations come in asynchronously in online streaming environments. It makes subspace tracking more efficient and less computationally expensive on extremely long data sequences [91]. Since ST can recover subspaces from incomplete frame vectors, it has the potential to further improve efficiency by downsampling the input frames [63]. The general formulation for the ST problem is as follows:

13 $$\begin{aligned} m_t = \sum _{x=1}^X k_x = l_t+s_t+e_t \quad for\; t=1,2,\dots ,n;\; X\in {1,2,3} \end{aligned}$$

where $m_t$ is input frame data at time *t*, and $l_t$, $s_t$, $e_t$ are low-rank, sparse, and noise components of $m_t$. The number of *k* is determined according to different decomposition forms, and the constraint conditions and approximate approximations on each component are determined according to different optimization methods.

#### Low-rank representation

Low-rank representation (LRR) can also be called low-rank optimization or low-rank minimization. Other unclassified models can be regarded as LRR. LRR is a minimization problem in mathematics. In LRR, the cost function measures the fit between the input matrix *M* and the approximation matrix *L* [63]. The mathematical formulation of LRR problem is as follows:

14 $$\begin{aligned} \min \, \Vert M-\hat{M}\Vert _F\quad \text {s.t.}\;rank(L)\le r \end{aligned}$$

where *M* is the input matrix, $\hat{M}$ the approximate matrix, $\Vert .\Vert _F$ the Frobenius norm, and *r* the rank. The basic form of LRR is similar to other models; therefore, most of the other unclassified models can be regarded as a category in LRR. For instance, RPCA and NMF can be obtained by similar architectures. Constraints other than rank constraints can be added for specific applications. LRR can be extended into an explicit or stable form by adding constraints on the sparse and noise components.

#### The extension to tensor

In DLSM framework, only some of the single-dimensional information is used when images are pretreated into data matrix *M* as vectors. This means that some multidimensional information is not taken into account in the process of decomposition. To improve the results, the tensor decomposition is proposed.

#### Tensor DLSM

**Fig. 4:**
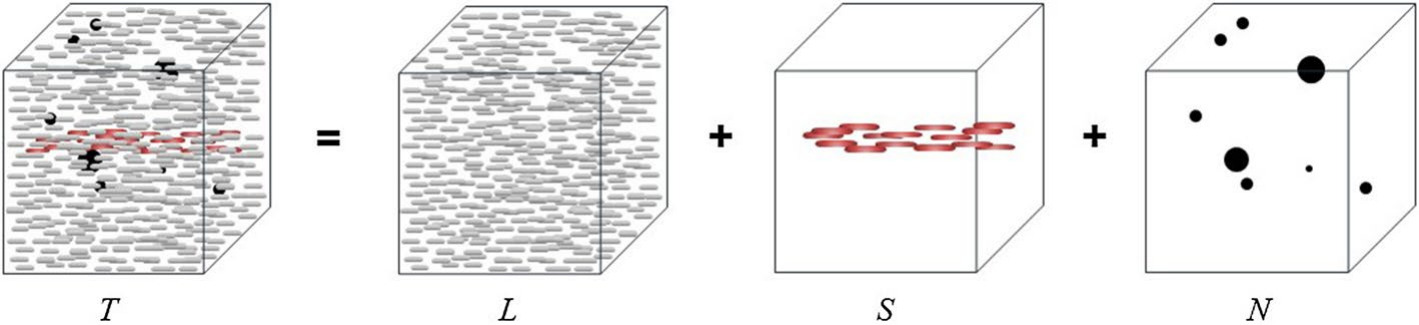
The illustration of tensor decomposition

When it comes to tensor, the most intuitive idea is to change all matrices to tensors directly since a tensor can be seen as a combination of several matrices. It is very similar to DLSM framework which subjects to $T=L+S+E$. The tensor DLSM extends all components to a tensor form as Fig. 4.

15 $$\begin{aligned} T=L+S+E \end{aligned}$$

where *T*, *L*, *S* and *N* represent the data tensor, low-rank tensor, sparse tensor and noise tensor, respectively. Similar to the matrix DLSM framework, it can be optimized and solved by a minimization problem. Some other classic matrix decomposition optimization methods have also been extended to tensor. The tensor robust principal component method [92] has been proposed based on tensor singular value decomposition (t-SVD) [93]. It has been demonstrated the effectiveness of image denoising. Another robust low-rank tensor recovery model based on RPCA has also been published for complex multilinear data analysis [94]. Rank sparsity tensor decomposition (RSTD) [95] and some other ideas based on stable principal component pursuit (PCP) also have been utilized in image processing.

#### Tensor decomposition

**Fig. 5:**
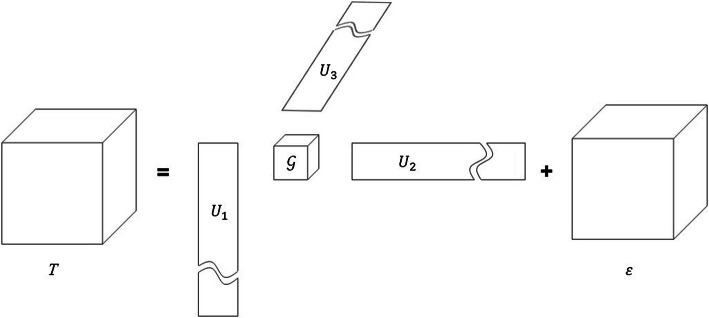
The illustration of Tucker decomposition

There are two classical tensor decomposition forms which are CANDECOMP/PARAFAC (CP) decomposition and Tucker decomposition [96]. Given a tensor $T\in \mathfrak {R}^{t_1\times t_2\times \cdots t_n}$, the CP decomposition and Tucker decomposition can be modeled as follows:

- Tucker decomposition
  16 $$\begin{aligned} T=g\times \prod _{i=1}^N U_i+\varepsilon \end{aligned}$$

where $g\in \mathfrak {R}^{r_1\times r_2\times \cdots r_n}$ is the core tensor, *r* is the rank of factor matrix $U_i\in \mathfrak {R}^{t_i\times r_i}$, and $\varepsilon$ represents the residuals. Figure 5 is a schematic representation of the Tucker decomposition. The Tucker decomposition is usually regarded as a non-convex optimization problem [63]. Two most famous and widely used solvers for Tucker decomposition are Tucker-ALS based on alternating least squares [96] and Tucker-ADAL based on alternating direction augmented Lagrangian [94]. SVD based on Tucker decomposition is generally called high-order singular value decomposition (HOSVD) [97, 98], which calculates the singular values of the three expansions $U_1,U_2,U_3$ of a three-dimensional tensor under Tucker decomposition. HOSVD-based ultrasound clutter optimization has been proposed [52, 99] and proved to be more robust to low sampling rates than SVD.

**Fig. 6:**
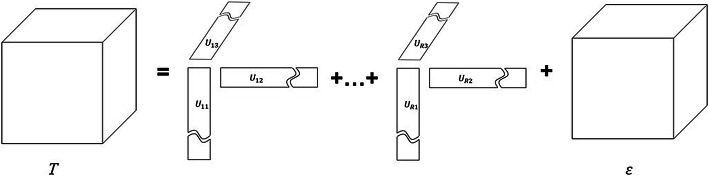
The illustration of CANDECOMP/PARAFAC (CP) decomposition

- CP decomposition
  17 $$\begin{aligned} T=U_1\circ U_2 \cdots \circ U_R+\varepsilon \end{aligned}$$

where *R* is the number of the components, $U_i\in \mathfrak {R}^{t_i\times r_i}$, $\varepsilon$ represents the residuals, and $U_1\circ U_2 \cdots \circ U_R$ is the CP model [71]. Figure 6 is a schematic representation of the CP decomposition. CP decomposition is similar in form to Tucker decomposition since the number of components in the factor matrices is the same [96]. The original CP problem is *NP*-hard. Therefore, the Frobenius norm is generally used to relax the low-rank constraint. Similar to Tucker decomposition, CP decomposition problem can also be solved by alternating least squares, called CP-ALS. To the best of our knowledge, there is currently no well-known article applying CP decomposition to ultrasound clutter filtering.

### Minimization problems

The decomposition problems generally turn into minimization problems or optimization problems in its original form or its Lagrangian form [63].

18 $$\begin{aligned} \min _{K_i} \, \sum _{i=1}^{x}{\lambda _if_i(K_i)}\quad \text {s.t.} \;C_i \end{aligned}$$

where $\lambda _i$ are the regularization parameters, $f_i(.)$ are the loss functions for low-rank, sparse, and noise components, $C_i$ are the constraints on $K_i$. Consistent with the decomposition problems, the minimization problems can be divided into three categories according to the number of constraints and loss functions imposed.

- $x=1$ is the case of implicit decomposition: $\min _L \, \lambda _1f_1(L) \quad \text {s.t.} \; C_1$
  where $C_1$ is $\Vert M-L\Vert _2=0$ or other forms. For sparse decomposition, the low-rank components are replaced by sparse components. This problem can be *NP*-hard, non-convex, or under specific constraints. Therefore, other formats of the loss functions are applied to relax the constraints when the problem is *NP*-hard or non-convex. For example, the loss function *f* is *rank* loss function in original MC model as $\min _{L}\, rank(L) \quad \text {s.t.} \; \Vert M-L\Vert _2=0$.
- $x=2$ is the case of explicit decomposition: $\min _{L,S} \; \lambda _1f_1(L)+\lambda _2f_2(S) \quad \text {s.t.} \; C_2$
  where $C_2$ is $\Vert M-L-S\Vert _2=0$ or other forms. For example, the $f_1$ and $f_2$ loss functions are *rank* and $l_0-norm$ loss functions in original RPCA model as $\min _{L,S}\, rank(L)+\lambda \Vert S\Vert _{l_0} \quad \text {s.t.} \;\Vert M-L-S\Vert _2=0$.
- $x=3$ is the case of stable decomposition: $\min _{L,S} \, \lambda _1f_1(L)+\lambda _2f_2(S)+\lambda _3f_3(N) \quad \text {s.t.} \; C_3$
  where $C_3$ is $\Vert M-L-S-E\Vert _2=0$ or other forms. For example, the $f_1$ and $f_2$ loss functions are *rank* and $l_0-norm$ in original RPCA model as $\min _{L,S}\, rank(L)+\lambda \Vert S\Vert _{l_0} \quad \text {s.t.} \; \Vert M-L-S\Vert _2=0$. The stable decomposition is generally adding constraints on the noise component based on the robust decomposition. The Frobenius norm loss function ($\lambda \Vert M-L-S\Vert _F^2=0$) is used in most cases.

Although there are some algorithms that can solve non-convex problems through mathematical approximation [100], in general, non-convex problems are difficult to solve with weak convergence. This is also an important role that minimization problems play.

### Loss functions

The loss function can be seen as a constraint of the decomposed matrices. In DLSM framework, loss functions are used on the minimization matrices as norm formats. For example, in implicit decomposition, explicit decomposition, and stable decomposition, the functions *f*(*S*), *f*(*L*), *f*(*E*), represent the loss functions or norms on sparse component, low-rank component, and noise component, respectively. However, in most cases, the original loss function will be replaced by other forms of the loss function to optimize and solve the problem. The common loss function forms (or norm forms) can be listed as follows:

- $l_0 \,$ norm loss function $(\Vert M\Vert _{l_0})$ is the number of non-zero entries.
- $l_1 \,$norm loss function $(\Vert M\Vert _{l_1} = \sum _{i,j}|M_{i,j}|)$ is the Manhattan distance.
- $l_2 \,$norm loss function $(\Vert M\Vert _{l_2} = \sqrt{\sum _{i,j}M^2_{i,j}})$ is also called the Frobenius norm ($l_{F} \,$norm loss function $(\Vert M\Vert _{l_{F}} = \sqrt{\sum _{i,j}M^2_{i,j}})$ ).
- $l_{\infty } \,$norm loss function $(\Vert M\Vert _{l_{\infty }} = max_{i,j}|M_{i,j}|)$ is also called the max norm ($(\Vert M\Vert _{max} = max_{i,j}|M_{i,j}|)$).
- $l_{*} \,$norm loss function $(\Vert M\Vert _{l_{*}})$ is the sum of singular values.

### Solvers

The models are solved by specific algorithms, which are called solvers in DLSM [63, 71] framework. Solvers are generally applied to the models after the minimization problem has been optimized and the loss function has been relaxed. Solvers can be broadly divided into regularization-based approaches and statistical-based approaches [101]. As for regularization approaches, the data matrices are regularized by convex surrogates with different features [63]. Typical regularization approaches include singular value thresholding (SVT) [102], accelerated proximal gradient (APG) [103], and augmented Lagrange multiplier (ALM) [83]. In terms of statistical-based approaches, prior distributions are used to capture low-rank or sparse properties and predict the joint distribution of the measured entries and unknown variables. Meanwhile, posterior distributions of the unknown variables can be approximated by Bayesian-based methods [63].

Although many solvers are proposed to solve the optimization problems under DLSM framework, most of the mainstream algorithms for ultrasound clutter suppression are based on SVD. SVD-based clutter suppression algorithms that are proposed and reviewed [19, 20, 22, 38, 43] based on traditional CFI before 2011. In these algorithms, SVD is used as one of the steps or iterations within many of the algorithms we evaluated. After 2015, with the rapid development of ultrasound technologies like plane-wave ultrasound, SVD was combined with ultrafast plane-wave imaging, which can provide a huge amount of data at a high frame rate, to improve the effectiveness of SVD and overcome the limitation of low frame rate [18, 51, 104, 105]. Due to the excellent performance of SVD on large datasets, SVD-based clutter suppression algorithms based on the plane-wave ultrasound have become a popular and mainstream research area. The SVD-based algorithms have been used in functional ultrasound [106, 107], super resolution ultrasound localization microscopy [104, 108] and high-sensitivity microvessel perfusion imaging [18, 51] due to its excellent performance in the ultrasound clutter suppression and the microvascular imaging [21].

Due to the obvious disadvantages of SVD, DLSM framework contains many approximate SVD and non-SVD algorithms for higher efficiency and lower computational cost, which have the potential for real-time ultrasound clutter suppression.

## Experiments

DLSM framework has been successfully utilized to video surveillance, face recognition, texture modeling, video inpainting, audio separation, and latent semantic indexing [109]. However, only a few algorithms under DLSM framework have been applied to ultrasound clutter suppression. Herein, we apply DLSM algorithms as the clutter filter for CFI. To that end, we test if DLSM algorithms can be used for clutter suppression and conduct simulation experiments, phantom experiments, and in vivo experiments. Finally, we will conclude a list of algorithms that are suitable for ultrasound clutter suppression.

### Experiment data

Three datasets are used in this experiment which are simulation data, phantom data, and in vivo rat data. For each dataset, raw RF-data, complex envelope data, and B-mode data formats are used for analysis. The specific parameters and obtaining process of three datasets and a brief introduction of three data formats are given in the following subsections.

#### Simulation data

**Fig. 7:**
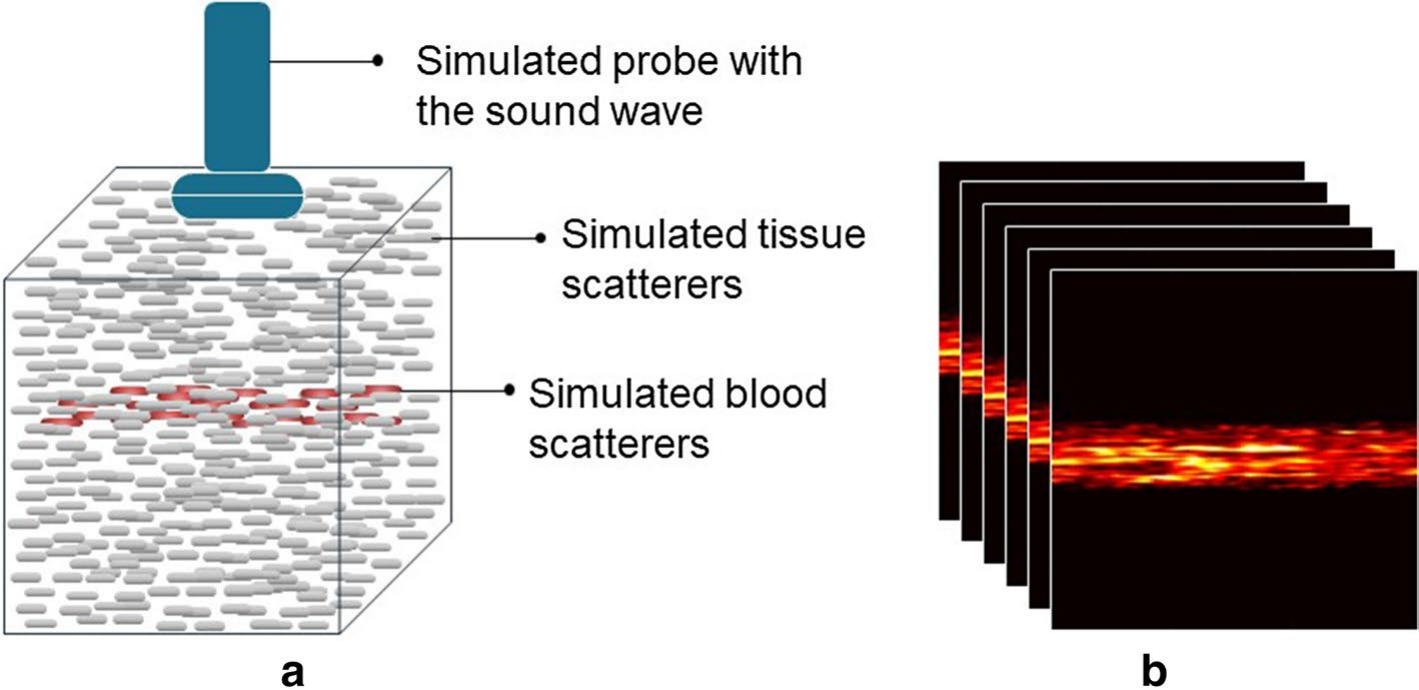
The illustration of the simulation data. **a** The simulation cube with tissue scatterers and blood scatterers. The red blood scatterers are in the middle and moving to the right. The simulated sound waves focus in the center. **b** A series of simulation data frames obtained from simulation experiments

The simulation data include a set of ultrasound simulation frames as Fig. 7 shows. The ultrasound simulation data are generated by the Field II simulation program implemented in MATLAB [110, 111]. A cube $A\in \mathfrak {R}^{60 \times 60 \times 60 }$ is built to represent the tissue filled with scatterers given the fact that each voxel is $1\, \text{mm}^3$. A vessel through the middle of the cube with a radius of $20\, \text{mm}$ is generated by scatterers flowing to the right. The max velocity in the center of the vessel is $15\, \text{mm}/\text{s}$. Assuming sound waves travel from the top to the bottom and focus on the center. Probe frequency and sampling frequency are set to $7.27\, \text{mHz}$ and $40\, \text{mHz}$, respectively. The frame rate is set to $1000\, \text{fps}$ and 64 active elements are used for beamforming.

#### Phantom data

**Fig. 8:**
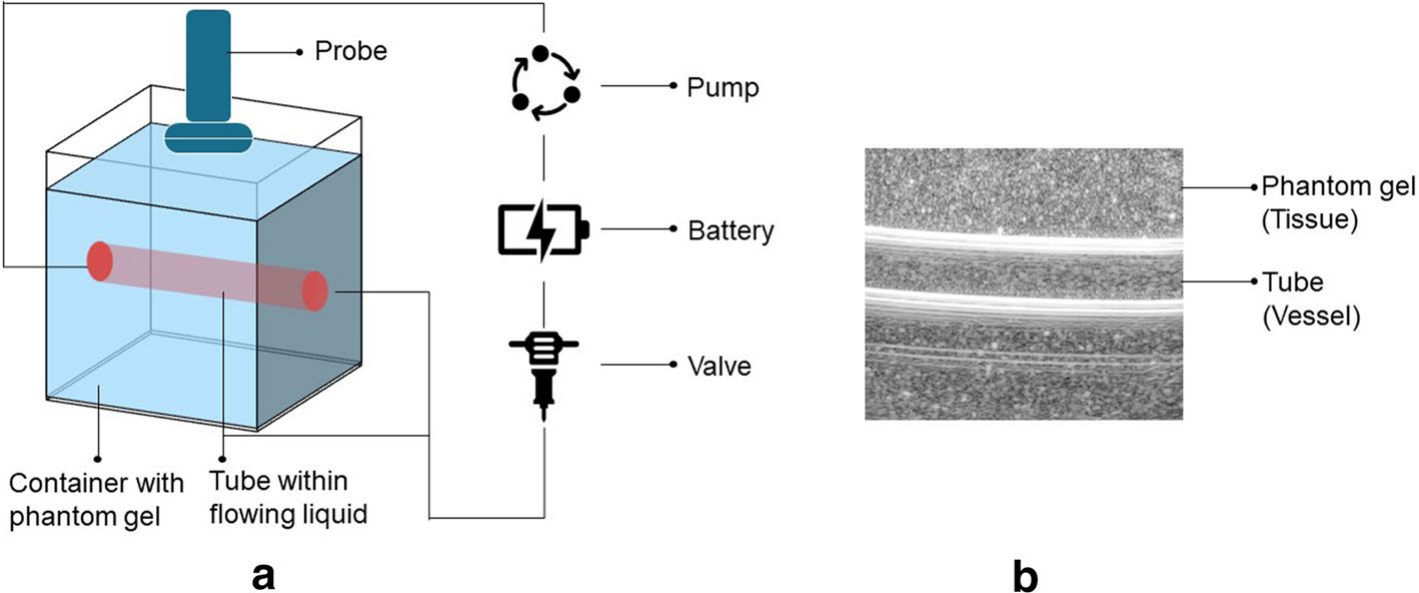
The illustration of the phantom experiments. **a** The illustration of phantom data collection experiment. **b** The B-mode image of the first frame in phantom data

The phantom was created to simulate a cube of tissue including one blood vessel which travels across the cube in the middle. Knox unflavored gelatin, water, and sugar-free Metamucil psyllium fiber supplement were gently heated and mixed to prepare the phantom gel which represents soft tissue. An intra-venous tube simulating a venous structure model runs through the gel cube. Probe frequency and sampling frequency are set to 10 MHz and 40 MHz, respectively. The Alpinion E-Cube R12 ultrasound system is used in ultrasound data collection with an L3-12H linear array probe. Figure 8 briefly illustrates the phantom experiment.

#### Rat data

**Fig. 9:**
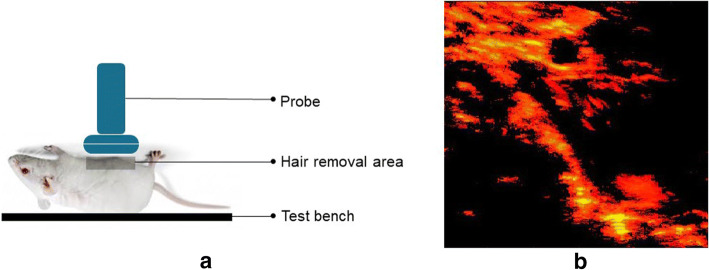
The illustration of the in vivo rat experiments. **a** The illustration of the in vivo rat data collection experiment. **b** A schematic representation of sparse component of the in vivo rat data

The acquisition of the rat data was under the supervision of the Animal Care Facility of Concordia University. A 27-week-old Sprague–Dawley male rat was anesthetized for ultrasound scanning. The experiment followed the guidelines of the Canadian Council on Animal Care and was approved by the Animal Ethics Committee of Concordia University $(\#30000259)$. The probe frequency and the sampling frequency were set to 10 MHz and 40 MHz, respectively. Similarly, as with phantom data, the Alpinion E-Cube R12 research ultrasound system with an L3-12H linear array probe was used. The schematic diagram of the in vivo rat experiment is shown in Fig. 9.

#### Data formats

Both real and simulated ultrasound data are available in three formats, which are raw RF data, complex envelope data, and B-mode data. Common ultrasound probes generally consist of a piezoelectric transducer array that emits and receives signals. The backscatter signal which is processed by the preamplifier and the time gain compensation is referred as radio-frequency (RF) signal. The RF signal then processed by an envelope detector becomes complex envelope data. Last, the complex envelope data are log-compressed into a grayscale format. And the data are further passed through intensity mapping and post-processing filtering. The final readable image is commonly called brightness mode (B-mode) image. RF frames generally have a very large size since the sampling rate of the RF data is usually extremely high. This high sampling rate is not necessary for envelope data as it does not have high-frequency contents. Therefore, envelope and B-mode images can be downsampled by a large factor. RF data may also be downsampled by a small factor, but the Nyquist sampling rate should be considered to avoid aliasing.

### Experiment methods

In “Decomposition into low-rank and sparse matrices (DLSM) framework” section, DLSM framework is introduced and built as Fig. 3 shows. The DLSM algorithms are classified in four groups which are implicit decomposition, explicit decomposition, stable decomposition, and tensor decomposition. In this experiment, all algorithms are selected from LRSLibrary [61, 63, 71] which provides a group of low-rank and sparse matrix decomposition algorithms in moving object detection. In LRSLibrary, these algorithms are further subdivided into robust PCA (RPCA), subspace tracking (ST), matrix completion (MC), three-term decomposition (TTD), low-rank representation (LRR), nonnegative matrix factorization (NMF), nonnegative tensor factorization (NTF), and standard tensor decomposition (TD) according to the models. Due to the flexible conversion between models and their similar mathematical formulations, in this paper, TTD can be a subcategory in stable PCA under stable decomposition. Similarly, NTF belongs to the subcategory under the tensor decomposition (TD) model.

In the first step, the DLSM algorithms are applied to three formats of simulation data to verify the performance of all algorithms compared to sparse component with ground truth and give a computing time contrast. Then, all algorithms are used on phantom data to find out if DLSM suits ultrasound data with real ultrasound features. In the third step, rat data are used for verification and comparison. The acquired data have three formats which are RF data format, complex envelope data format, and B-mode data format. The results of different data formats and different datasets are grouped for comparison to find the optimal conditions of ultrasound clutter suppression.

All experiments are processed by a normal desktop computer with an i7-4770 CPU @ 3.40 GHz and 16.0 GB RAM.

### Evaluation metrics

Two main indicators are used to evaluate the performance of various algorithms, which are signal-to-noise ratio (SNR) [112] and contrast-to-noise ratio (CNR). The SNR and CNR are calculated as follows:

19 $$\begin{aligned} \text{SNR}=\frac{\mu _1}{\sigma _1},\quad \text{CNR}=\frac{|\mu _1-\mu _2|}{\sqrt{\sigma _1^2+\sigma _2^2}/2} \end{aligned}$$

where $\mu _1$ and $\sigma _1$ are the mean intensity value and the standard deviation of the background window, $\mu _2$ and $\sigma _2$ are the mean intensity value and the standard deviation of the target window.

### Experiment results

The results of 106 DLSM algorithms on three datasets and their three formats are reported in this section. The results of all algorithms include the SNR, CNR, calculation times, and images for visual observation. Since all the output images are sparse components of the same data and are very similar, we classify the results according to their performance and report the number of algorithms in each category instead of SNR and CNR of all algorithms.

The results of all algorithms are divided into several categories. The results which fall in the first category are considered to be good results as they give the correct sparse matrix with a pure blank background which means high robustness to noise and dynamic background and strong decomposition ability. The cases when the output sparse component is more than 100 times higher than background pixel values are also regarded as good results. The results which fall in the second category are considered to be defective. These results either contain background noise which is supposed to be part of the low-rank components, or are noisy and algorithms failed to decompose. The results in the third category are not considered because some algorithms failed to run due to some limitations like non-negative limitations or real input limitations. Algorithms with this type of results are called restricted algorithms in this section.

The information that all algorithms, including their model classifications, are from LRSLibrary [61, 63, 71], and they have all been proved to be successfully applied to moving object detection on traffic video.

#### Simulation experiments

The experiments first applied simulation data to verify the availability and approximate performance of all algorithms. Meanwhile, the computation cost and time of these algorithms on ultrasound clutter suppression are also tested. The first experiment applied all 106 DLSM algorithms to the RF simulation data. Among 106 algorithms, 11 of them were out of memory and failed to run. These algorithms cannot deal with the large size of simulation data because they use the full singular value decomposition or QR decomposition and require a huge memory to initialize (7.9 GB). Meanwhile, there are 6 algorithms that require non-negative input and cannot take RF data as input. Consequently, a total of 17 of these two kinds of algorithms are classified as restricted algorithms.

In terms of the remaining 89 algorithms, only 19 of them are able to output relatively pure sparse components that match the ground truth without any processes of RF simulation data. To be precise, only three algorithms (abbreviation: LRR-ROSL, RPCA-IALM, RPCA-IALM-BLWS) give a truly pure background as zero matrices (all entries in sparse matrices except the ones presenting simulated vessel are 0). The other 16 results highlight simulated vessel with a non-zero background. Since the value of the background pixels is 1000–10,000 times less than the value of sparse component, we consider it to be a pure result without low-rank components. The possible reason is the particular small values of RF simulation data and low dynamic range. In general, these results with the CNR values above 1.6 are classified as good results in Table 2. The results of the other 44 algorithms are very noisy with the CNR values less than 1.1. As for these algorithms, the sparse parts in simulation data are not clearly determined and the clutter is not well suppressed. The remaining 24 algorithms give blank output due to low dynamic range and other reasons. Almost all the DLSM algorithms give an SNR of about 0.759, so SNR is not reported in detail here.

**Table 2:** The 19 algorithms with the CNR values above 1.6

Group	Abbreviation	Time	CNR	Group	Abbreviation	Time	CNR
RPCA	IALM*	0.590	1.681	MC	IALM-MC	6.537	1.680
RPCA	IALM-BLWS*	2.278	1.680	TTD	MAMR	1.861	1.740
LRR	ROSL*	0.359	1.688	NMF	PNMF	13.556	1.733
RPCA	DECOLOR	3.013	1.602	RPCA	PRMF	1.280	1.687
RPCA	EALM	9.068	1.677	RPCA	RegL1-ALM	3.634	1.686
RPCA	flip-SPCP-max-QN	71.933	1.688	MC	RPCA-GD	4.747	1.627
RPCA	flip-SPCP-sum-SPG	214.900	1.688	RPCA	SSGoDec	0.034	1.736
RPCA	GoDec	0.072	1.736	TD	Tucker-ADAL	6.131	1.736
RPCA	GreGoDec	0.199	1.736	TD	Tucker-ALS	0.101	1.736
TD	HoSVD	4.461	1.736				

The algorithms with * give pure background. The remaining algorithms are arranged in alphabetical order of abbreviations

Due to the extremely small data values and dynamic ranges, a large number of algorithms are invalidated. Therefore, in the second step, the order of magnitude and dynamic range of RF simulation data are expanded to re-examine the performance of all algorithms.

After processing RF data, 56 algorithms show good results. Among these algorithms, 16 of them give correct sparse components with a zero-valued background, others give sparse components 1000–10,000 times greater than background pixel values. The results of the remaining 33 algorithms are noisy. These algorithms either do not correctly isolate sparse components or contain inseparable background noise with similar values. Most good results have a CNR greater than 1.3, while noisy results generally have a CNR less than 1. Similarly, almost all the DLSM algorithms with good results give an SNR of about 0.759. There are a few good results with a CNR less than 1. The algorithms with such results only highlight the sparsest parts which reduce the mean intensity values of the target window. However, these results are considered to be good because the unhighlighted sparse components still have higher intensities than backgrounds. The results after increasing dynamic range are listed in Table 3. Examples of different kinds of results in simulation exp

**Table 3:** The 16 algorithms with pure background after increasing dynamic range

Group	Abbreviation	Time	CNR	Group	Abbreviation	Time	CNR
RPCA	IALM*	0.604	1.681	RPCA	FPCP	0.102	1.392
RPCA	IALM-BLWS*	1.647	1.680	RPCA	FW-T	0.647	0.611
LRR	ROSL*	0.408	1.688	TD	HoRPCA-S-NCX	116.955	1.689
RPCA	APG	4.155	1.667	RPCA	Lag-SPCP-QN	0.517	0.377
RPCA	APG-PARTIAL	3.559	1.661	RPCA	Lag-SPCP-SPG	0.955	0.354
RPCA	AS-RPCA	1.890	1.682	TD	OSTD	0.663	0.479
NMF	DRMF	2.580	1.640	RPCA	PCP	27.078	1.677
RPCA	DUAL	100.797	1.682	RPCA	SVT	453.337	1.682

**Fig. 10:**
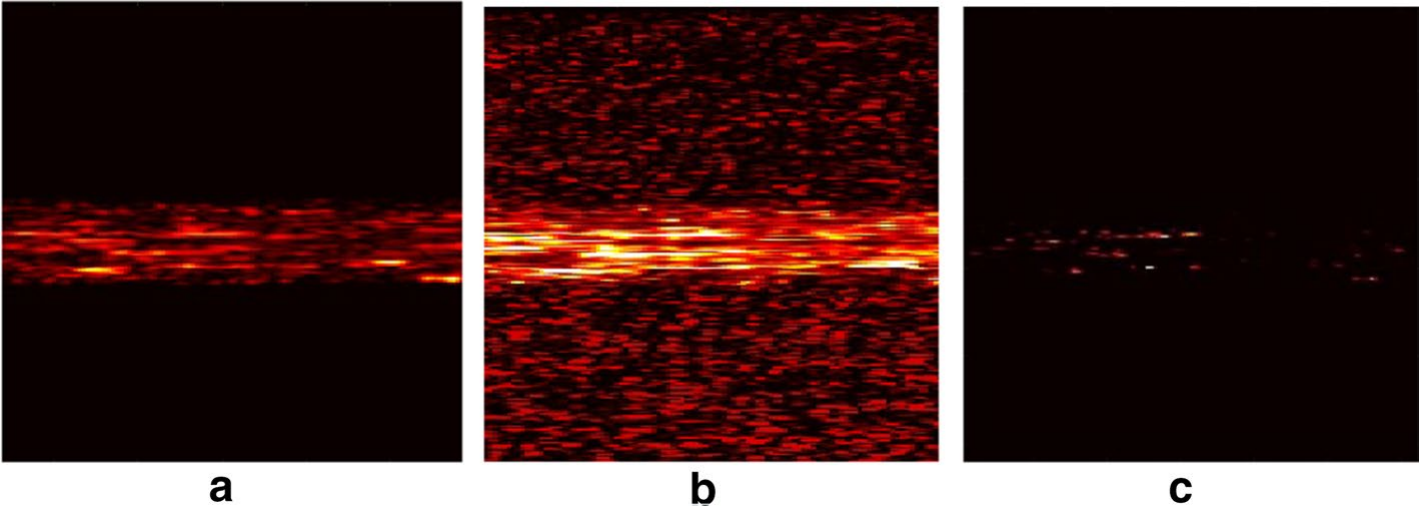
The output result images of simulation data. **a** The output of sparse component obtained by the IALM algorithm on original simulated RF data. It is a typical good result representing correct decomposition and pure sparse components. **b** The output of sparse component obtained by the ADM algorithm on original simulated RF data. It is a typical noisy result with background noise as sparse components. **c** The output of sparse component obtained by the OSTD algorithm on processed simulated RF data with larger dynamic range. The algorithms with a CNR less than 1 in Table 3 give such results with pure background because they only show the sparsest parts

The algorithms with * give pure background on original data. The remaining algorithms are arranged in alphabetical order of abbreviations

eriments are shown in Fig. 10.

The complex envelope simulation data are obtained by Hilbert transform based on the RF data. For this reason, the complex envelope data do not have the problem of miniature pixel values and low dynamic range. However, the SNR and CNR of the complex envelope simulation data are lower than the SNR and CNR of RF simulation data. Except for the 11 algorithms that are limited by frame size, 24 of the remaining algorithms show good results. In addition, 13 algorithms are affected by complex numbers generated by the Hilbert transform and thus failed to run. The results of other algorithms are noisy. After enlarging the dynamic range of the complex envelope simulation data, 8 algorithms which failed on original simulation data give good results on the preprocessed data. These algorithms are sensitive to the changes of dynamic ranges. The results on complex envelope simulation data are shown in Table 4. Obviously, the CNR in the results of complex envelope simulation data is far less than the CNR on the RF data.

**Table 4:** The algorithms with good results on complex envelope simulation data

Group	Abbreviation	Time	CNR	Group	Abbreviation	Time	CNR
				TTD	3WD	5.061	0.079
				RPCA	ALM	19.662	0.049
NMF	Deep-Semi-NMF	0.169	0.049	NMF	Deep-Semi-NMF	0.221	0.049
LRR	EALM	10.096	1.723	LRR	EALM	0.580	0.049
NMF	ENMF	42.921	0.049	NMF	ENMF	45.057	0.049
RPCA	flip-SPCP-max-QN	358	0.151	RPCA	flip-SPCP-max-QN	294	0.151
RPCA	flip-SPCP-sum-SPG	403	0.151	RPCA	flip-SPCP-sum-SPG	630	0.151
RPCA	FPCP*	0.138	0.154	RPCA	FPCP	0.181	0.049
RPCA	GoDec	0.116	0.049	RPCA	GoDec	0.127	0.049
RPCA	GreGoDec	0.396	0.049	RPCA	GreGoDec	0.430	0.092
TD	HoRPCA-S-NCX*	201	0.059	TD	HoRPCA-S-NCX*	210	0.059
TD	HoSVD	3.083	0.049	TD	HoSVD	3.074	0.049
				LRR	IALM	3.999	0.049
MC	IALM-MC	10.481	0.051	MC	IALM-MC	10.784	0.051
NMF	iNMF	1.675	0.040	NMF	iNMF	1.916	0.040
				RPCA	Lag-SPCP-QN*	77.200	0.176
				RPCA	Lag-SPCP-SPG*	92.931	0.186
MC	LMaFit	0.512	0.071	MC	LMaFit	0.547	0.071
NMF	NeNMF	0.141	0.049	NMF	NeNMF	0.158	0.049
NMF	nmfLS2	0.512	0.049	NMF	nmfLS2	0.563	0.049
NMF	NMF-MU	3.206	0.049	NMF	NMF-MU	3.379	0.049
NMF	NMF-PG	0.431	0.049	NMF	NMF-PG	164	0.032
RPCA	noncvxRPCA	1.044	0.048	RPCA	noncvxRPCA	0.193	0.089
NMF	PNMF	24.802	0.048	NMF	PNMF	25.377	0.048
				RPCA	R2PCP*	2.251	0.058
LRR	ROSL*	1.018	0.058	LRR	ROSL*	1.039	0.058
NMF	Semi-NMF	0.210	0.030	NMF	Semi-NMF	2.305	0.029
RPCA	SSGoDec	3.772	0.049	RPCA	SSGoDec	3.729	0.051
				RPCA	TFOCS-EC	26.941	0.132
				RPCA	TFOCS-IC	26.162	0.094
TD	Tucker-ADAL	10.290	0.049	TD	Tucker-ADAL	458	0.039
TD	Tucker-ALS	0.217	0.049	TD	Tucker-ALS	0.216	0.049
RPCA	VBRPCA	4.031	0.046	RPCA	VBRPCA	6.471	0.069

The results on original data are listed in the left column and the results on processed data are listed in the right column. The algorithms with * give pure background. The algorithms are arranged in alphabetical order of abbreviations

The third step of the simulation experiment is using B-mode data. As for the results of B-mode simulation data, 40 DLSM algorithms have successfully detected the simulate vessel on original B-mode simulation data. Meanwhile, 12 algorithms are affected by high peak values in the background and keep static peaks into sparse components. These algorithms give pure sparse matrices after suppressing peak values. After enlarging the dynamic range of the original B-mode data, another 10 algorithms successfully detect correct sparse components. Therefore, 62 algorithms can successfully separate the correct sparse components. The other algorithms which give very noisy results may need parameter adjustment and threshold process. The results of simulation experiment on B-mode data are reported in Table 5.

**Table 5:** The algorithms with good results on B-mode simulation data

Group	Abbreviation	Time	CNR	Group	Abbreviation	Time	CNR
TTD	3WD$\circ$	2.027	1.486	RPCA	Lag-SPCP-QN*	2.809	0.494
LRR	ADM	0.563	3.401	RPCA	Lag-SPCP-SPG*	8.961	0.456
RPCA	ALM$\bullet$	18.748	1.827	MC	LMaFit	0.424	1.889
RPCA	APG$\circ$*	4.229	1.855	MC	LRGeomCG	0.811	1.885
RPCA	APG-PARTIAL$\circ$*	3.696	1.860	RPCA	LSADM$\circ$	1.454	1.847
RPCA	AS-RPCA	2.180	1.803	TTD	MAMR	1.642	1.781
RPCA	DECOLOR	3.450	1.717	NMF	ManhNMF	1.422	1.903
NMF	Deep-Semi-NMF	0.195	1.903	RPCA	MoG-RPCA	1.710	1.934
NMF	DRMF$\circ$*	2.461	1.842	NMF	NeNMF	0.073	1.903
RPCA	DUAL$\circ$*	89.410	1.824	NMF	NMF-ALS	1.848	1.903
LRR	EALM$\bullet$	0.321	1.903	NMF	NMF-ALS-OBS	1.987	1.903
RPCA	EALM$\circ$	4.324	1.840	NMF	nmfLS2	0.206	1.903
NMF	ENMF	9.056	1.903	NMF	NMF-MU	1.643	1.903
LRR	FastLADMAP	0.769	1.903	NMF	NMF-PG	32.465	1.899
RPCA	flip-SPCP-max-QN	102.000	1.835	RPCA	noncvxRPCA	0.100	1.903
RPCA	flip-SPCP-sum-SPG	230.000	1.835	RPCA	NSA1$\bullet$	0.255	1.902
MC	FPC	34.877	1.442	TD	OSTD$\bullet$*	0.764	1.451
RPCA	FPCP*	0.150	1.875	RPCA	PCP$\circ$*	9.978	1.842
RPCA	FW-T$\circ$*	0.722	0.370	NMF	PNMF	13.424	1.903
RPCA	GA$\bullet$	0.028	1.904	RPCA	PRMF	1.336	1.857
RPCA	GoDec	0.096	1.903	RPCA	R2PCP$\bullet$*	1.269	2.024
RPCA	GreGoDec	0.282	1.903	RPCA	RegL1-ALM	3.918	1.833
TD	HoRPCA-S-NCX	112.090	1.836	TTD	RMAMR$\bullet$	5.369	1.561
TD	HoSVD	4.493	1.903	LRR	ROSL*	0.369	1.830
LRR	IALM	1.880	1.903	MC	RPCA-GD$\circ$	4.946	1.891
RPCA	IALM$\circ$*	0.701	1.840	NMF	Semi-NMF	0.134	1.331
RPCA	IALM-BLWS$\circ$*	1.800	1.843	RPCA	SSGoDec	1.206	1.903
MC	IALM-MC	5.729	1.848	RPCA	TFOCS-EC$\bullet$	6.388	1.903
NMF	iNMF	1.148	1.770	TD	Tucker-ADAL	74.711	1.903
RPCA	L1F$\bullet$	1.022	0.817	TD	Tucker-ALS	0.118	1.903
LRR	LADMAP	0.446	1.903	RPCA	VBRPCA	0.306	0.343

The algorithms with $\circ$ are affected by high peak values and get good results after suppressing peaks. The algorithms with $\bullet$ only get good results after increasing the dynamic range. The algorithms with * give pure background

#### Phantom experiments

The next set of experimental data used for testing is phantom data. The phantom data are used to test whether DLSM framework is suitable for ultrasound clutter suppression with real ultrasound noise and other ultrasound features. The phantom data also consist of three formats, which are RF phantom data, complex envelope phantom data, and B-mode phantom data. Except for 11 inapplicable algorithms due to size limitation, the other 95 algorithms have a huge difference in computing time ranging from less than 0.1 s to more than 500 s.

As for RF phantom data, the order of magnitude of all pixels is first adjusted into the range of $10^{\pm 3}$. However, the structured peak pixels that are caused by bright structure generated at the rebound reflection interface still affect many algorithms. 36 algorithms clearly show the simulated vessel with a pure background with an average CNR of 3.5. Meanwhile, 43 algorithms only highlight bright edges as the sparse components with an average CNR of 0.4. The structured peak pixels of RF phantom data can compromise the calculation of some algorithms when these bright edges have a pixel value $10^3$ times larger than the remaining pixel values. Therefore, the peak values are processed logarithmically to achieve the gray balance and reduce the dynamic range. After logarithmic processing, 22 additional algorithms are able to display sparse components correctly excluding bright and static edges. Among them, 19 algorithms are previously affected by peaks, and 3 algorithms are defective on the original data. The results of remaining algorithms are still noisy, and parameter adjustment should be applied to these algorithms for better performance. The results of RF phantom experiments are shown in Table 6.

**Table 6:** The algorithms with good results in RF phantom experiments

Group	Abbreviation	Time	CNR	Group	Abbreviation	Time	CNR
TTD	3WD$\bullet$	1.826	2.393	RPCA	Lag-SPCP-SPG	31.118	2.626
TTD	ADMM$\bullet$	4.009	2.478	MC	LMaFit	0.260	2.651
RPCA	ALM	5.456	2.672	MC	LRGeomCG	0.817	2.640
RPCA	APG$\bullet$	5.585	2.747	RPCA	LSADM$\bullet$	1.443	2.747
RPCA	APG-PARTIAL$\bullet$	4.690	2.747	TTD	MAMR	2.263	2.681
RPCA	AS-RPCA	2.697	2.732	RPCA	MoG-RPCA	9.156	2.777
RPCA	DECOLOR	10.266	4.895	NMF	nmfLS2	0.219	2.672
NMF	Deep-Semi-NMF	0.150	2.672	RPCA	NSA1$\bullet$	1.549	2.746
NMF	DRMF$\bullet$	2.723	2.754	RPCA	NSA2$\bullet$	1.656	2.746
RPCA	DUAL$\bullet$	215	2.746	MC	OptSpace$\bullet$	7.020	2.526
LRR	EALM	0.351	2.672	MC	OR1MP$\bullet$	0.089	2.627
RPCA	EALM$\bullet$	37.360	2.744	TD	OSTD$\bullet$	70.747	1.799
RPCA	flip-SPCP-max-QN$\bullet$	119	2.768	RPCA	PCP$\bullet$	26.791	2.745
RPCA	flip-SPCP-sum-SPG$\bullet$	431	2.768	NMF	PNMF	16.963	2.684
RPCA	FPCP	0.108	2.672	RPCA	PRMF	1.573	2.623
RPCA	FW-T$\circ$	0.591	2.578	RPCA	R2PCP	2.241	2.703
RPCA	GA	0.031	3.652	RPCA	RegL1-ALM	4.745	2.774
RPCA	GM	0.155	2.775	TTD	RMAMR	9.728	2.545
RPCA	GoDec	0.097	2.674	LRR	ROSL	0.421	2.715
ST	GRASTA$\circ$	1.394	1.207	MC	RPCA-GD	6.215	2.622
RPCA	GreGoDec	0.237	2.821	TD	RSTD$\circ$	91.200	1.636
TD	HoRPCA-S-NCX	70.134	2.777	MC	ScGrassMC	4.093	2.567
TD	HoSVD	0.497	2.672	NMF	Semi-NMF	1.267	2.295
RPCA	IALM	0.796	2.748	RPCA	SSGoDec	1.496	2.736
LRR	IALM$\bullet$	2.003	2.672	MC	SVP$\bullet$	3.235	2.471
RPCA	IALM-BLWS$\bullet$	2.474	2.748	RPCA	TFOCS-EC	9.815	2.188
MC	IALM-MC	7.764	2.419	RPCA	TFOCS-IC	9.568	2.197
RPCA	L1F	2.680	0.837	TD	Tucker-ADAL	267	2.617
RPCA	Lag-SPCP-QN	15.766	2.684	TD	Tucker-ALS	0.123	2.672

The algorithms with $\circ$ are the 3 new algorithms work on processed data, which are defective on original data. The algorithms with $\bullet$ are sensitive to structured peak pixels and work after logarithmic processing

The complex envelope phantom data are then used for experiments. The number of good results of the complex envelope phantom data is less than the number of good results of RF phantom data. 26 algorithms successfully detected the simulated vessel and 33 algorithms only showed bright edges, which is an intra-venous (IV) tube representing the vessel wall. The CNR of all results is less than 0.4. After suppressing the edge brightness logarithmically, 11 of these algorithms that have been affected by edges can separate pure sparse components. It shows that the extremely high bright structures can affect the sensitivity to sparse components in many algorithms. However, there are still some algorithms that give noisy results. At the same time, 19 algorithms cannot take complex numbers as input. The results on complex envelope phantom data are shown in Table 7.

**Table 7:** The algorithms with good results on complex envelope phantom data

Group	Abbreviation	Time	CNR	Group	Abbreviation	Time	CNR
TTD	3WD$\bullet$	4.947	0.032	RPCA	Lag-SPCP-SPG	38.770	0.118
RPCA	ALM$\bullet$	86.748	0.070	MC	LMaFit	0.441	0.063
RPCA	APG$\bullet$	14.096	0.064	MC	MC-NMF	1.733	0.056
RPCA	APG-PARTIAL$\bullet$	19.703	0.064	NMF	NeNMF	0.179	0.070
NTF	bcuNTD	23.042	0.065	NMF	nmfLS2	0.787	0.070
NMF	Deep-Semi-NMF	0.275	0.070	NMF	NMF-MU	4.429	0.070
NMF	DRMF$\bullet$	2.467	0.251	RPCA	noncvxRPCA$\circ$	0.239	0.070
LRR	EALM$\bullet$	113.071	0.070	RPCA	NSA1$\bullet$	3.560	0.065
NMF	ENMF	56.960	0.070	RPCA	NSA2$\bullet$	3.704	0.064
RPCA	flip-SPCP-max-QN	194.004	0.110	RPCA	PCP$\bullet$	29.737	0.064
RPCA	flip-SPCP-sum-SPG	774.004	0.110	NMF	PNMF	32.414	0.072
RPCA	FPCP	0.156	0.069	RPCA	R2PCP$\circ$	1.410	0.071
RPCA	GoDec	0.164	0.071	LRR	ROSL	1.077	0.070
RPCA	GreGoDec	0.603	0.070	NMF	Semi-NMF	0.184	0.078
MC	GROUSE*	2.090	0.123	RPCA	SSGoDec	4.876	0.071
TD	HoRPCA-S-NCX	174.635	0.064	RPCA	TFOCS-EC$\bullet$	29.885	0.052
TD	HoSVD	2.527	0.070	TD	Tucker-ADAL	654.740	0.010
LRR	IALM$\bullet$	6.495	0.070	TD	Tucker-ALS	0.269	0.070
MC	IALM-MC	15.723	0.055	RPCA	VBRPCA$\circ$	20.913	0.077
RPCA	Lag-SPCP-QN	27.778	0.079	NMF	NMF-PG*	34.973	0.063

The algorithms with $\circ$ are the 3 new algorithms work on processed data, which are defective on original data. The algorithms with $\bullet$ are sensitive to structured peak pixels and work after logarithmic processing. Two algorithms with * get good results on original envelope data but are defective on processed data

**Fig. 11:**
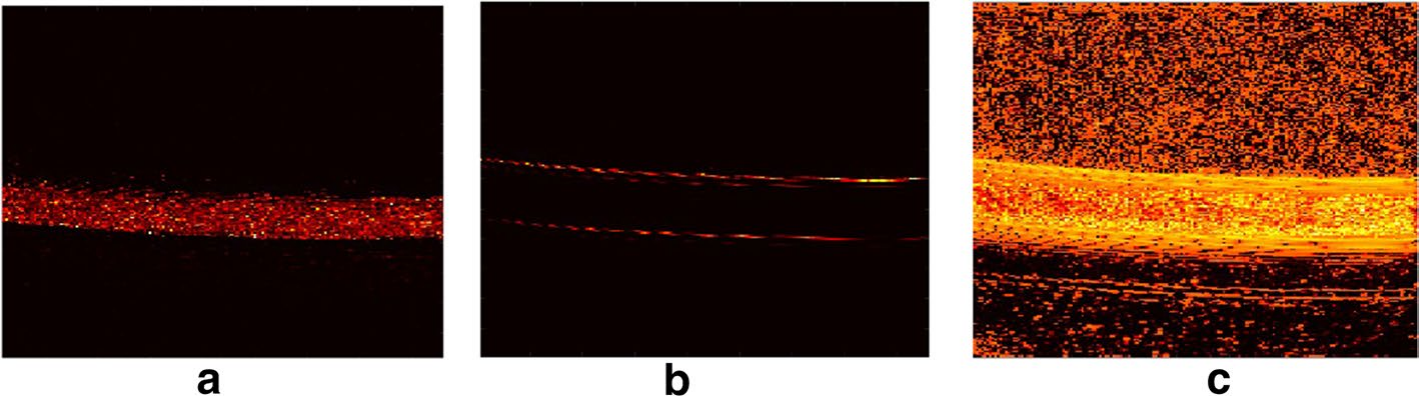
Three typical output results of phantom experiments. **a** A typical good result showing pure sparse components without noise. This image is obtained by ALM algorithm on original phantom data. **b** A typical output affected by bright edge structures. This image is obtained by APG algorithm on original phantom data. Because the pixel values of bright edges are 1000 times larger than the pixel values in the rest of the image, the flow sparse component in the middle of the tube cannot be observed. **c** A typical noisy result showing sparse components with indivisible noise. This image is obtained by RSTD algorithm on original phantom data

The last format of data to be applied is B-mode data. As for B-mode phantom data, 49 algorithms successfully give good results with a CNR higher than 2. However, the results of four of these algorithms contain bright edges which are considered to be low-rank components. The results of 35 algorithms only show the edges. Among them, a few sensitive algorithms can also partly detect sparse partition with obvious motion. However, only several bright pixels with motion can be detected. After reducing the dynamic range, no algorithm is affected by edges and 75 algorithms give sparse components with pure background. Examples of good results and noisy results in phantom experiments are shown in Fig. 11. The results of B-mode phantom data are shown in Table 8.

**Table 8:** The algorithms with good results on B-mode phantom data

Group	Abbreviation	Time	CNR	Group	Abbreviation	Time	CNR
RPCA	LSADM$\bullet$	1.455	3.582	MC	RPCA-GD$\bullet$	6.118	3.165
RPCA	L1F	2.595	1.038	MC	ScGrassMC	4.123	1.338
RPCA	DECOLOR	7.015	2.847	LRR	EALM$\bullet$	10.899	3.681
RPCA	RegL1-ALM	4.352	3.700	LRR	IALM$\bullet$	2.469	3.681
RPCA	GA$\circ$	0.032	3.680	LRR	ADM**	0.668	0.024
RPCA	GM$\circ$	0.153	3.713	LRR	LADMAP	0.363	3.681
RPCA	MoG-RPCA	4.691	3.359	LRR	FastLADMAP	0.802	3.681
RPCA	noncvxRPCA$\bullet$	0.110	3.681	LRR	ROSL	0.421	3.712
RPCA	NSA1$\bullet$	1.407	3.602	TTD	3WD$\bullet$	1.942	2.964
RPCA	NSA2$\bullet$	1.537	3.568	TTD	MAMR	2.784	3.154
RPCA	flip-SPCP-sum-SPG	276	3.695	TTD	RMAMR	6.776	2.289
RPCA	flip-SPCP-max-QN	138	3.695	TTD	ADMM$\circ$*	3.627	0.794
RPCA	Lag-SPCP-SPG*	5.010	1.598	NMF	NMF-MU	2.143	3.681
RPCA	Lag-SPCP-QN*	7.219	0.685	NMF	NMF-PG	8.774	3.565
RPCA	FW-T*	0.715	3.073	NMF	NMF-ALS	2.406	3.681
RPCA	BRPCA-MD$\bullet$	283	3.724	NMF	NMF-ALS-OBS	2.710	3.681
RPCA	BRPCA-MD-NSS$\bullet$	291	3.511	NMF	PNMF	16.815	3.681
RPCA	VBRPCA	4.627	3.692	NMF	ManhNMF	2.292	3.662
RPCA	PRMF	1.522	3.522	NMF	NeNMF	0.066	3.681
RPCA	TFOCS-EC$\bullet$	9.131	3.349	NMF	LNMF**	0.204	0.279
RPCA	GoDec	0.095	3.681	NMF	ENMF	13.546	3.681
RPCA	SSGoDec	1.459	3.679	NMF	nmfLS2	0.320	3.681
RPCA	GreGoDec	0.229	3.681	NMF	Semi-NMF	0.154	2.604
ST	GRASTA	1.321	1.156	NMF	Deep-Semi-NMF	0.156	3.681
MC	FPC	49.672	2.454	NMF	iNMF	1.482	3.650
MC	GROUSE**	1.580	0.068	NMF	DRMF$\bullet$*	2.461	3.497
MC	IALM-MC	6.992	3.690	TD	HoSVD	0.532	3.681
MC	LMaFit	0.314	3.300	TD	HoRPCA-S-NCX	89.622	3.693
MC	LRGeomCG	0.757	3.723	TD	Tucker-ADAL	258	3.573
MC	MC-NMF$\circ$	0.585	3.423	TD	Tucker-ALS	0.130	3.681
MC	OR1MP$\circ$	0.096	3.365				

The algorithms with $\circ$ are the 3 new algorithms work on processed data, which are defective on original data. The algorithms with $\bullet$ are sensitive to structured peak pixels and work after logarithmic processing. Three algorithms with ** get good results on original envelope data but are defective on processed data. The algorithms with * give pure backgrounds

#### In vivo experiments

In the third step, rat data are used to test the performance of these algorithms on real ultrasound data with small vessels-like tissues. The RF rat data, complex envelope rat data, and B-mode rat data are used to be compared.

**Fig. 12:**
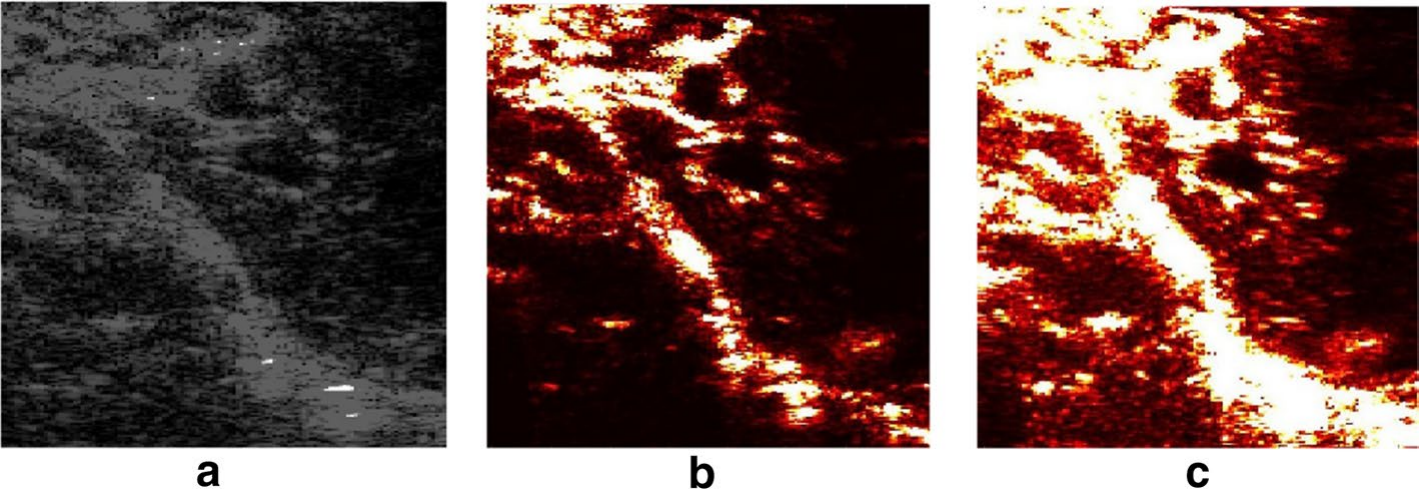
The examples of the results of rat experiments. **a** The B-mode image of rat data for comparison. **b** is obtained by ALM algorithm on original rat data. The dynamic background and noise are filtered out relatively well. **c** is obtained by APG algorithm on original rat data. Large areas of dynamic tissue are classified as sparse components. Since there is no ground truth for in vivo rat data, the results are described using relatively good and relatively noisy

In terms of RF rat data, 82 algorithms give very good and similar results, and 7 algorithms show noisy and meaningless results. The other 17 algorithms are restricted due to the size limitation or non-negative limitation. The complex envelope rat data remains to share similar results with RF rat data. As for B-mode rat data, 92 algorithms successfully detected vessel-like tissues and only 3 algorithms failed to show any part of the sparse components. Examples of good results and noisy results in in vivo rat experiments are shown in Fig. 12. Since most algorithms give results with similar SNR and CNR, the evaluation of results combine subjective observations and numerical analysis. Due to the unknown in vivo structure, we lack ground truth for the accuracy of the assessment results. Only algorithms with pure backgrounds are shown in Table 9 due to similar results and limited space.

**Table 9:** The algorithms with pure backgrounds on in vivo data

Group	Abbreviation	CNR
RF in vivo data		
TTD	ADMM	0.306
Envelope in vivo data		
RPCA	Lag-SPCP-SPG	0.258
RPCA	R2PCP	0.153
NMF	DRMF	0.510
B-mode in vivo data		
RPCA	R2PCP	0.416
RPCA	Lag-SPCP-QN	0.519
RPCA	Lag-SPCP-SPG	0.502
TTD	ADMM	0.453
TD	RSTD	0.449
TD	OSTD	0.521

## Discussion

A total of 106 algorithms were tested in this paper. Analyzing the results obtained from simulation, phantom, and in vivo experiments, we found that 11 algorithms require huge memory (7.9 GB for frame size $250\times 125$, 20 frames) due to the singular value decomposition or QR decomposition process. Since typical ultrasound frames are large in size, the left unitary matrix in full singular value decomposition demands an excessive amount of memory, e.g., ADM. There are two possible solutions to this problem. First, the approximate SVD can be calculated and stored in every iteration instead of full SVD [55, 113]. Second, small overlapping patches from the ultrasound frames can be considered to formulate the data matrix which will substantially reduce the size of the Casorati matrix and eventually the memory footprint. Another advantage of using this windowing technique is that it can automatically equalize uneven noise distribution by normalizing the power locally [51]. The 11 algorithms with size limitation are listed in Table 10.

**Table 10:** The 11 algorithms with size limitation

Group	Abbreviation	Algorithm name
RPCA	IALM-LMSVDS	IALM with LMSVDS
RPCA	ADM	Alternating direction method
ST	GOSUS	Grassmannian online subspace updates with structured-sparsity
ST	pROST	Robust PCA and subspace tracking from incomplete observations using L0-surrogates
ST	ReProCS	Provable dynamic robust PCA or robust subspace tracking
ST	MEDRoP	Memory efficient dynamic robust PCA
MC	PG-RMC	Nearly optimal robust matrix completion
MC	MC-logdet	Top-N recommender system via matrix completion
MC	OP-RPCA	Robust PCA via outlier prsuit
MC	SVT	A singular value thresholding algorithm for matrix completion
TD	t-SVD	Tensor SVD in Fourier domain

Moreover, there are 6 algorithms that require non-negative input. Since ultrasound RF data usually contain both positive and negative values, these algorithms are not suitable for working with RF data for clutter suppression. 20 algorithms giving good results on RF simulation data are tested with the absolute value of RF data to confirm the impact of non-negative requirements on ultrasound clutter suppression. Although all of these 53 algorithms are still capable of showing show high-contrast vessel structures, the SNR obtained with absolute value RF data (0.81) is slightly greater than the original SNR (0.76) showing a significant increase of background noise in sparse components. At the same time, the CNR obtained with absolute value RF data (1.69) is slightly lower than the original CNR (1.73), which proves that nonnegative requirement has only limited effect on the accuracy of the DLSM decomposition. The 6 algorithms with size limitations are listed in Table 11.

**Table 11:** The algorithms with non-negative requirement

Group	Abbreviation	Algorithm name
MC	MC-NMF	Nonnegative mtrix completion
NMF	NMF-MU	NMF solved by mltiplicative udates
NMF	NMF-ALS-OBS	NMF solved by alternating least squares with optimal brain surgeon
NMF	LNMF	Spatially localized NMF
NMF	iNMF	Incremental subspace learning via NMF
TD	CP-APR	PARAFAC/CP decomposition solved by alternating Poisson regression

Another type of restricted algorithms is affected by complex inputs. From the experiment results, it is obvious that complex envelope data are not suitable for ultrasound clutter suppression since it takes longer calculation time and gives poor performance. Also, 13 algorithms are affected by complex value and cannot separate low-rank and sparse components well. Among them, 13 algorithms cannot take complex numbers as input, and some algorithms are stuck in a longer loop that requires more than 300 s. Algorithms which failed due to complex numbers are listed below in Table 12. In addition, the extremely small CNR obtained from the envelope data is only one-hundredth of the ones obtained from other datasets which indicates that envelope data are not suitable as an input form of ultrasound clutter suppression.

**Table 12:** The 13 algorithms that cannot take complex numbers as input

Group	Abbreviation	Algorithm nme
RPCA	DECOLOR	Contiguous outliers in the low-rank representation
RPCA	MoG-RPCA	Mixture of Gaussians RPCA
RPCA	FW-T	SPCP solved by Frank–Wolfe method
MC	LRGeomCG	Low-rank matrix completion by Riemannian optimization
MC	RPCA-GD	Robust PCA via gradient descent
LRR	ADM	Alternating direction method
LRR	LADMAP	Linearized ADM with adaptive penalty
LRR	FastLADMAP	Fast LADMAP
TTD	MAMR	Motion-assisted matrix restoration
TTD	RMAMR	Robust motion-assisted matrix restoration
TD	HoRPCA-IALM	HoRPCA solved by IALM
TD	HoRPCA-S	HoRPCA with singleton model solved by ADAL
TD	RSTD	Rank sparsity tensor decomposition

Among the remaining algorithms, 17 algorithms are easily affected by outliers. These algorithms cannot denoise the peak values when the dynamic range is roughly greater than $11.5\, \text{bits}$, which is the natural logarithm of difference between maximum and minimum. These 17 algorithms have performed well on pre-processed data and showed good results in simulation experiments and phantom experiments. However, they are not robust to outliers. In the simulation experiments, these algorithms divided the background peak pixels into sparse components, resulting in a noisy background. Similarly, they are not robust to large-shaped structured outliers and divide bright static edges into sparse components in phantom experiments. These 17 algorithms that are susceptible to outliers are listed in Table 13. Since complex envelope data are not suitable for ultrasound clutter suppression, the performance of the algorithms on complex envelope data has not been considered.

**Table 13:** The algorithms not robust to the outliers

Group	Abbreviation	Algorithm name
RPCA	PCP	Principal component pursuit
RPCA	IALM-BLWS	IALM with BLWS
RPCA	APG-PARTIAL	Partial accelerated proximal gradient
RPCA	APG	Accelerated proximal gradient
RPCA	DUAL	Dual RPCA
RPCA	LSADM	LSADM
RPCA	GA	Grassmann average
RPCA	GM	Grassmann median
RPCA	NSA1	Non-smooth augmented Lagrangian v1
RPCA	NSA1	Non-smooth augmented Lagrangian v2
RPCA	FW-T	SPCP solved by Frank–Wolfe method
RPCA	TFOCS-EC	TFOCS with equality constraints
LRR	EALM	Exact ALM
LRR	IALM	Inexact ALM
TTD	3WD	3-way-decomposition
NMF	DRMF	Direct robust matrix factorization
TD	OSTD	Online stochastic tensor decomposition

In addition, a pure background (0 dB) is of great significance for vascular image segmentation and process and analysis of other medical images [46, 114]. However, this is a difficult goal due to the probe jitter, dynamic backgrounds, noise, shadows, and many other reasons. Therefore, only a few results have pure background on simulation data and phantom data. Furthermore, no result has pure background on in vivo rat data because of the complex tissue motions and the harsh conditions. Some algorithms have a strong ability dealing with these challenges and give pure backgrounds on simulation data and phantom data. These algorithms are listed in Table 14.

**Table 14:** The algorithms with the potential to give a pure background

Group	Abbreviation	Algorithm name
RPCA	PCP	Principal component pursuit
RPCA	FPCP	Fast PCP
RPCA	R2PCP	Riemannian robust principal component pursuit
RPCA	IALM	Inexact ALM
RPCA	IALM-BLWS	IALM with BLWS
RPCA	APG-PARTIAL	Partial accelerated proximal gradient
RPCA	APG	Accelerated proximal gradient
RPCA	DUAL	Dual RPCA
RPCA	Lag-SPCP-SPG	Lagrangian SPCP solved by spectral projected gradient
RPCA	Lag-SPCP-QN	Lagrangian SPCP solved by Quasi-Newton
RPCA	FW-T	SPCP solved by Frank–Wolfe method
LRR	ROSL	Robust orthonormal subspace learning
NMF	DRMF	Direct robust matrix factorization
TD	HoRPCA-S-NCX	HoRPCA with singleton model solved by ADAL (non-convex)
TD	OSTD	Online stochastic tensor decomposition

Overall, in terms of calculation time, DLSM algorithms take the longest time to run complex envelope data in comparison with RF data and B-mode data. Due to its large amount of calculations, complex envelope data take twice as long as RF data do to run. This confirms again that complex envelope data are not suitable for ultrasound clutter suppression. Meanwhile, RF data require slightly less computation time than B-mode data. This may be caused by the extra information RF data contain. Meanwhile, we can find that DLSM algorithms use a slightly longer time on preprocessed data than on original data. However, the algorithms separate sparse components more accurately. Table 15 lists the average time taken by the fastest 20 algorithms on different datasets and different data formats.

**Table 15:** The average time taken by the fastest 20 algorithms

	RF data (s)	Complex envelope data (s)	B-mode data (s)
Original simulation data	0.19	0.67	0.28
Original phantom data	0.31	0.58	0.30
Original rat data	0.31	0.50	0.29
Preprocessed simulation data	1.05	1.21	0.30
Preprocessed phantom data	0.69	2.18	0.33
Preprocessed rat data	0.77	1.81	0.61

The experimental results prove that ultrasound data are very different from ordinary video surveillance frames. All DLSM algorithms can be successfully applied to surveillance images. However, some of them are not suitable for ultrasound data. There are quite a few algorithms that are not suitable for ultrasound RF data and complex envelope data, which may due to the complexity of the RF data and the complex space of complex envelope data. The simulation experiments prove that some algorithms are still not robust to ultrasonic clutter and are not sensitive to the data with overall small pixel values ($<10^{-3}$). As for these algorithms, the low-rank components of the results often contain inseparable background flicker, noise, and tiny motion. These algorithms have been listed in Table 13. Meanwhile, the phantom experiment results prove that some DLSM algorithms are not robust and stable with a high dynamic range greater than $10\, \text{bits}$. For ultrasound data, an area with small values often exists in a uniform tissue. Edges that are much brighter than other tissues are also common due to the strong reflections at the interface. The CNR after preprocessing the ultrasound data is generally higher than the CNR of raw data. The result of the data that removed the peak is also significantly better than the results of raw data. Therefore, it is necessary to preprocess the ultrasound image when applying the DLSM algorithm. Moreover, parameter adjustment or other math improvements are necessary when applying some DLSM algorithms on ultrasound data to get the best filtering performance.

On the other hand, in terms of ultrasound data formats, experiments show that B-mode ultrasound data can make more algorithms successful for vascular detection. The B-mode ultrasound data may lose information. However, the outliers that may affect DLSM algorithms may also be weakened by Hilbert transform an absolute process. This might be the reason why more DLSM algorithms work for B-mode data. Although B-mode data has more good results than RF data, RF data require slightly less average calculation time and are more suitable for real-time requirements. The algorithms in Table 16 are relatively stable in all three datasets. These algorithms all require less than 1 s for computation while giving the correct sparse components. Experiments show that they may be more suitable for ultrasound clutter suppression.

**Table 16:** The algorithms require less than 1 s calculation time

Group	Abbreviation	Algorithm name
LRR	ADM	Alternating direction method
LRR	LADMAP	Linearized ADM with adaptive penalty
LRR	FastLADMAP	Fast LADMAP
LRR	ROSL	Robust orthonormal subspace learning
MC	GROUSE	Grassmannian rank-one update subspace estimation
MC	LMaFit	Low-rank matrix fitting
MC	LRGeomCG	Low-rank matrix completion by Riemannian optimization
NMF	nmfLS2	Nonnegative matrix factorization with sparse matrix
NMF	Semi-NMF	Semi-nonnegative matrix factorization
NMF	Deep-semi-NMF	deep semi-nonnegative matrix factorization
RPCA	FPCP	Fast PCP
RPCA	L1F	L1 filtering
RPCA	noncvxRPCA	Robust PCA via nonconvex rank approximation
RPCA	VBRPCA	Variational Bayesian RPCA
RPCA	GoDec	Go decomposition
RPCA	GreGoDec	Greedy semi-soft GoDec algorithm
TD	Tucker-ADAL	Tucker decomposition solved by ADAL
TD	Tucker-ALS	Tucker decomposition solved by ALS

Finally, 22 algorithms which are most robust to noise with the best performance in all the experiments are listed in Table 17. These algorithms may have a strong ability for ultrasound clutter suppression.

**Table 17:** The most robust algorithms with the best performance

Group	Abbreviation	Algorithm name
RPCA	FPCP	Fast PCP
RPCA	L1F	L1 filtering
RPCA	DECOLOR	Contiguous outliers in the low-rank representation
RPCA	RegL1-ALM	Low-rank matrix approximation under robust L1-norm
RPCA	MoG-RPCA	Mixture of Gaussians RPCA
RPCA	Lag-SPCP-SPG	Lagrangian SPCP solved by spectral projected gradient
RPCA	Lag-SPCP-QN	Lagrangian SPCP solved by Quasi-Newton
RPCA	PRMF	Probabilistic robust matrix factorization
RPCA	GoDec	Go Decomposition
RPCA	SSGoDec	Semi-soft GoDec
RPCA	GreGoDec	Greedy semi-soft GoDec algorithm
MC	IALM-MC	Inexact ALM for matrix completion
MC	LMaFit	Low-rank matrix fitting
MC	LRGeomCG	Low-rank matrix completion by Riemannian optimization
LRR	ROSL	Robust orthonormal subspace learning
TTD	MAMR	Motion-assisted matrix restoration
NMF	PNMF	Probabilistic nonnegative matrix factorization
NMF	nmfLS2	Nonnegative matrix factorization with sparse matrix
NMF	Semi-NMF	Semi-nonnegative matrix factorization
NMF	Deep-Semi-NMF	Deep semi-nonnegative matrix factorization
TD	HoSVD	High-order singular value decomposition
TD	HoRPCA-S-NCX	HoRPCA with singleton model solved by ADAL (nonconvex)
TD	Tucker-ADAL	Tucker decomposition solved by ADAL
TD	Tucker-ALS	Tucker decomposition solved by ALS

In this paper, we adapted different techniques originally proposed for natural images in the field of computer vision for ultrasound color flow imaging. As ultrasound images have unique characteristics due to the physics of sound propagation, these images have the so-called “speckle noise”. We believe that the results of this paper can be generalized to other imaging modalities that are affected by diffraction, such as optical coherence tomography (OCT).

## Conclusion

The performance of 106 established low-rank and sparse decomposition algorithms for clutter filtering has been tested in this work. Our results show that few robust matrix decomposition techniques are suitable for solving the limitations of SVD-based ultrasound clutter suppression methods such as sensitivity to large noise. In addition, several matrix decomposition techniques show the potential for real-time implementation on commercial ultrasound machines due to their low computational complexity. Furthermore, some preprocessing is necessary when applying this framework to ultrasound data. Finally, some of the algorithms studied in this work can automatically estimate the optimal power Doppler images without requiring extensive manual tuning, which may pave the way for easier commercial and clinical translation of ultrasound clutter suppression (Additional file 1).

## Supplementary information

**Additional file 1: Table S1.** Information and abbreviations of DLSM algorithms.

## Data Availability

The datasets generated and analyzed during the current study are available from http://data.sonography.ai/.

## Abbreviations

B-mode: Brightness-mode
CP: CANDECOMP/PARAFAC
CFI: Color flow imaging
CNR: Contrast-to-noise ratio
CTA: Computed tomography angiography
CT: Computed tomography
DLSM: Decomposition into low-rank and sparse matrices
DSA: Digital subtraction angiography
DUS: Duplex ultrasonography
FIR: Finite impulse response
IIR: Infinite impulse response
LRR: Low-rank representation
MRA: Magnetic resonance angiography
MRI: Magnetic resonance imaging
MC: Matrix completion
NMF: Non-negative matrix factorization
OCT: Optical coherence tomography
PCA: Principal component analysis
PCP: Principal component pursuit
RSTD: Rank sparsity tensor decomposition
RMC: Robust matrix completion
RNMF: Robust non-negative matrix factorization
RPCA: Robust principal component analysis
RPCP: Robust principal component pursuit
RST: Robust subspace tracking
SNR: Signal-to-noise ratio
SVD: Singular value decomposition
Stable NMF: Stable non-negative matrix factorization
Stable RPCA: Stable robust principal component analysis
ST: Subspace tracking
TD: Tensor decomposition
t-SVD: Tensor singular value decomposition
TTD: Three-term decomposition

## References

[1] Wang H, Naghavi M, Allen C, Barber RM, Bhutta ZA, Carter A, Casey DC, Charlson FJ, Chen AZ, Coates MM (2016). Global, regional, and national life expectancy, all-cause mortality, and cause-specific mortality for 249 causes of death, 1980–2015: a systematic analysis for the global burden of disease study 2015. Lancet.

[2] Dasgupta R, Fishman SJ. Issva classification. In: Seminars in pediatric surgery, vol. 23. Elsevier; 2014. p. 158–61. 10.1053/j.sempedsurg.2014.06.016 25241091

[3] Camici PG, Crea F (2007). Coronary microvascular dysfunction. N Engl J Med.

[4] Marinescu MA, Löffler AI, Ouellette M, Smith L, Kramer CM, Bourque JM (2015). Coronary microvascular dysfunction, microvascular angina, and treatment strategies. JACC Cardiovasc Imaging.

[5] McDonald DM, Baluk P. Significance of blood vessel leakiness in cancer. AACR. 2002. 12235011

[6] Padera TP, Stoll BR, Tooredman JB, Capen D, di Tomaso E, Jain RK (2004). Pathology: cancer cells compress intratumour vessels. Nature.

[7] Solomon SD, Chew E, Duh EJ, Sobrin L, Sun JK, VanderBeek BL, Wykoff CC, Gardner TW (2017). Diabetic retinopathy: a position statement by the american diabetes association. Diabetes Care.

[8] Association AD (2003). Peripheral arterial disease in people with diabetes. Diabetes Care.

[9] Dolan NC, Liu K, Criqui MH, Greenland P, Guralnik JM, Chan C, Schneider JR, Mandapat AL, Martin G, McDermott MM (2002). Peripheral artery disease, diabetes, and reduced lower extremity functioning. Diabetes Care.

[10] Healy D, Rogers P, Hii L, Wingfield M (1998). Angiongenesis: a new theory for endometriosis. Hum Reprod Update.

[11] Horsch AD, Weale AR. Imaging in vascular disease. Surgery. 2018.

[12] Evans DH, Jensen JA, Nielsen MB (2011). Ultrasonic colour Doppler imaging. Interface Focus.

[13] Demchuk AM, Menon BK, Goyal M (2016). Comparing vessel imaging: noncontrast computed tomography/computed tomographic angiography should be the new minimum standard in acute disabling stroke. Stroke.

[14] Rübenthaler J, Reiser M, Clevert D-A (2016). Diagnostic vascular ultrasonography with the help of color Doppler and contrast-enhanced ultrasonography. Ultrasonography.

[15] Bjaerum S, Torp H, Kristoffersen K (2002). Clutter filter design for ultrasound color flow imaging. IEEE Trans Ultrason Ferroelectr Freq Control.

[16] Oglat AA, Matjafri M, Suardi N, Oqlat MA, Abdelrahman MA, Oqlat AA (2018). A review of medical Doppler ultrasonography of blood flow in general and especially in common carotid artery. J Med Ultrasound.

[17] Gerhard-Herman M, Gardin JM, Jaff M, Mohler E, Roman M, Naqvi TZ (2006). Guidelines for noninvasive vascular laboratory testing: a report from the american society of echocardiography and the society for vascular medicine and biology. Vasc Med.

[18] Demené C, Deffieux T, Pernot M, Osmanski B-F, Biran V, Gennisson J-L, Sieu L-A, Bergel A, Franqui S, Correas J-M (2015). Spatiotemporal clutter filtering of ultrafast ultrasound data highly increases Doppler and fultrasound sensitivity. IEEE Trans Medical Imaging.

[19] Alfred C, Lovstakken L (2010). Eigen-based clutter filter design for ultrasound color flow imaging: a review. IEEE Trans Ultrason Ferroelectr Freq Control.

[20] Mauldin FW, Lin D, Hossack JA (2011). The singular value filter: a general filter design strategy for pca-based signal separation in medical ultrasound imaging. IEEE Trans Med Imaging.

[21] Solomon O, Cohen R, Zhang Y, Yang Y, He Q, Luo J, van Sloun RJ, Eldar YC. Deep unfolded robust pca with application to clutter suppression in ultrasound. IEEE Trans Med Imaging. 2019. 10.1109/TMI.2019.2941271 31535987

[22] Bjaerum S, Torp H, Kristoffersen K (2002). Clutter filters adapted to tissue motion in ultrasound color flow imaging. IEEE Trans Ultrason Ferroelectr Freq Control.

[23] Kadi AP, Loupas T (1995). On the performance of regression and step-initialized iir clutter filters for color Doppler systems in diagnostic medical ultrasound. IEEE Trans Ultrason Ferroelectr Freq Control.

[24] Hoeks A, Van de Vorst J, Dabekaussen A, Brands P, Reneman R (1991). An efficient algorithm to remove low frequency Doppler signals in digital Doppler systems. Ultrason imaging.

[25] Torp H (1997). Clutter rejection filters in color flow imaging: a theoretical approach. IEEE Trans Ultrason Ferroelectr Freq Control.

[26] Eckersley RJ, Chin CT, Burns PN (2005). Optimising phase and amplitude modulation schemes for imaging microbubble contrast agents at low acoustic power. Ultrasound Med Biol.

[27] Bruce M, Averkiou M, Tiemann K, Lohmaier S, Powers J, Beach K (2004). Vascular flow and perfusion imaging with ultrasound contrast agents. Ultrasound Med Biol.

[28] Simpson DH, Burns PN, Averkiou MA (2001). Techniques for perfusion imaging with microbubble contrast agents. IEEE Trans Ultrason Ferroelectr Freq Control.

[29] Hwang J-J, Simpson DH. Two pulse technique for ultrasonic harmonic imaging. Google Patents. US Patent 5,951,478. 1999.

[30] Simpson DH, Chin CT, Burns PN (1999). Pulse inversion Doppler: a new method for detecting nonlinear echoes from microbubble contrast agents. IEEE Trans Ultrason Ferroelectr Freq Control.

[31] Thomas L, Hall A. An improved wall filter for flow imaging of low velocity flow. In: 1994 Proceedings of IEEE ultrasonics symposium, vol. 3. New York: IEEE; 1994. p. 1701–4.

[32] Yoo YM, Managuli R, Kim Y (2003). Adaptive clutter filtering for ultrasound color flow imaging. Ultrasound Med Biol.

[33] Allam ME, Kinnick RR, Greenleaf JF (1996). Isomorphism between pulsed-wave Doppler ultrasound and direction-of-arrival estimation. II. Experimental results. IEEE Trans Ultrason Ferroelectr Freq Control.

[34] Vaitkus PJ, Cobbold RS, Johnston KW (1998). A new time-domain narrowband velocity estimation technique for Doppler ultrasound flow imaging. II. Comparative performance assessment. IEEE Trans Ultrason Ferroelectr Freq Control.

[35] Ledoux LA, Brands PJ, Hoeks AP (1997). Reduction of the clutter component in Doppler ultrasound signals based on singular value decomposition: a simulation study. Ultrasonic Imaging.

[36] Kargel C, Hobenreich G, Trummer B, Insana MF (2003). Adaptive clutter rejection filtering in ultrasonic strain-flow imaging. IEEE Trans Ultrason Ferroelectr Freq Control.

[37] Song F, Zhang D, Gong X (2006). Performance evaluation of eigendecomposition-based adaptive clutter filter for color flow imaging. Ultrasonics.

[38] Kruse DE, Ferrara KW (2002). A new high resolution color flow system using an eigendecomposition-based adaptive filter for clutter rejection. IEEE Trans Ultrason Ferroelectr Freq Control.

[39] Alfred C, Cobbold RS (2008). Single-ensemble-based eigen-processing methods for color flow imaging-part i. the hankel-svd filter. IEEE Trans Ultrason Ferroelectr Freq Control.

[40] Candes EJ, Sing-Long CA, Trzasko JD (2013). Unbiased risk estimates for singular value thresholding and spectral estimators. IEEE Trans Signal Process.

[41] Bar-Zion A, Tremblay-Darveau C, Solomon O, Adam D, Eldar YC (2016). Fast vascular ultrasound imaging with enhanced spatial resolution and background rejection. IEEE Trans Med Imaging.

[42] Kim M, Abbey CK, Hedhli J, Dobrucki LW, Insana MF (2017). Expanding acquisition and clutter filter dimensions for improved perfusion sensitivity. IEEE Trans Ultrason Ferroelectr Freq Control.

[43] Lovstakken L, Bjaerum S, Kristoffersen K, Haaverstad R, Torp H (2006). Real-time adaptive clutter rejection filtering in color flow imaging using power method iterations. IEEE Trans Ultrason Ferroelectr Freq Control.

[44] Mauldin FW, Viola F, Walker WF (2010). Complex principal components for robust motion estimation. IEEE Trans Ultrason Ferroelectr Freq Control.

[45] Gallippi CM, Nightingale KR, Trahey GE (2003). Bss-based filtering of physiological and arfi-induced tissue and blood motion. Ultrasound Med Biol.

[46] Bayat M, Fatemi M, Alizad A (2019). Background removal and vessel filtering of noncontrast ultrasound images of microvasculature. IEEE Trans Biomed Eng.

[47] Adabi S, Ghavami S, Fatemi M, Alizad A (2019). Non-local based denoising framework for in vivo contrast-free ultrasound microvessel imaging. Sensors.

[48] Olleros GG, Stuart MB, Jensen JA, Hoyos CAV, Hansen KL. Spatiotemporal filtering for synthetic aperture slow flow imaging. In: 2018 IEEE international ultrasonics symposium (IUS). New York: IEEE; 2018. p. 1–4.

[49] Bergqvist G, Larsson EG (2010). The higher-order singular value decomposition: theory and an application [lecture notes]. IEEE Signal Process Mag.

[50] Du Y, Zhang M, Alfred C, Yu W. Low-rank adaptive clutter filtering for robust ultrasound vector flow imaging. In: 2018 IEEE international ultrasonics symposium (IUS). New York: IEEE; 2018. p. 1–9.

[51] Song P, Manduca A, Trzasko JD, Chen S (2016). Ultrasound small vessel imaging with block-wise adaptive local clutter filtering. IEEE Trans Med Imaging.

[52] Kim M, Zhu Y, Hedhli J, Dobrucki LW, Insana MF (2018). Multidimensional clutter filter optimization for ultrasonic perfusion imaging. IEEE Trans Ultrason Ferroelectr Freq Control.

[53] Nayak R, Kumar V, Webb J, Gregory A, Fatemi M, Alizad A (2018). Non-contrast agent based small vessel imaging of human thyroid using motion corrected power Doppler imaging. Sci Rep.

[54] Baranger J, Arnal B, Perren F, Baud O, Tanter M, Demené C (2018). Adaptive spatiotemporal svd clutter filtering for ultrafast Doppler imaging using similarity of spatial singular vectors. IEEE Trans Med Imaging.

[55] Ashikuzzaman M, Belasso C, Kibria MG, Bergdahl A, Gauthier CJ, Rivaz H. Low rank and sparse decomposition of ultrasound color flow images for suppressing clutter in real-time. IEEE Trans Med Imaging. 2019. 10.1109/TMI.2019.2941865 31535988

[56] Candès EJ, Li X, Ma Y, Wright J (2011). Robust principal component analysis?. J ACM.

[57] Bayat M, Fatemi M. Concurrent clutter and noise suppression via low rank plus sparse optimization for non-contrast ultrasound flow Doppler processing in microvasculature. In: 2018 IEEE international conference on acoustics, speech and signal processing (ICASSP). New York: IEEE; 2018. p. 1080–4.

[58] Lin Z (2016). A review on low-rank models in data analysis. Big Data Inf Anal.

[59] Davenport MA, Romberg J (2016). An overview of low-rank matrix recovery from incomplete observations. IEEE J Sel Top Signal Process.

[60] Lauritzen SL (1996). Graphical models.

[61] Sobral A, Bouwmans T, Zahzah E-h. Lrslibrary: Low-rank and sparse tools for background modeling and subtraction in videos. Robust low-rank and sparse matrix decomposition: applications in image and video processing. 2016.

[62] Zhou X, Yang C, Zhao H, Yu W (2015). Low-rank modeling and its applications in image analysis. ACM Comput Surv.

[63] Bouwmans T, Sobral A, Javed S, Jung SK, Zahzah E-H (2017). Decomposition into low-rank plus additive matrices for background/foreground separation: a review for a comparative evaluation with a large-scale dataset. Comput Sci Rev.

[64] Chandrasekaran V, Sanghavi S, Parrilo PA, Willsky AS (2011). Rank-sparsity incoherence for matrix decomposition. SIAM J Optim.

[65] Li P, Yang X, Zhang D, Bian Z (2008). Adaptive clutter filtering based on sparse component analysis in ultrasound color flow imaging. IEEE Trans Ultrason Ferroelectr Freq Control.

[66] Chen G, Needell D (2016). Compressed sensing and dictionary learning. Finite Frame Theory Complet Introd Overcompleteness.

[67] Cevher V, Duarte MF, Hegde C, Baraniuk R. Sparse signal recovery using Markov random fields. In: Adv Neural Inf Process Syst. 2009. p. 257–64.

[68] Cevher Volkan, Sankaranarayanan Aswin, Duarte Marco F., Reddy Dikpal, Baraniuk Richard G., Chellappa Rama (2008). Compressive Sensing for Background Subtraction. Lecture Notes in Computer Science.

[69] Ramirez I, Sprechmann P, Sapiro G. Classification and clustering via dictionary learning with structured incoherence and shared features. In: 2010 IEEE computer society conference on computer vision and pattern recognition. New York: IEEE; 2010. p. 3501–3508.

[70] Tong T, Wolz R, Coupé P, Hajnal JV, Rueckert D, Initiative ADN (2013). Segmentation of mr images via discriminative dictionary learning and sparse coding: application to hippocampus labeling. NeuroImage.

[71] Sobral AC. Robust low-rank and sparse decomposition for moving object detection: from matrices to tensors. PhD thesis, Université de La Rochelle. 2017.

[72] Bouwmans T, Javed S, Zhang H, Lin Z, Otazo R (2018). On the applications of robust pca in image and video processing. Proc IEEE.

[73] Otazo R, Candes E, Sodickson DK (2015). Low-rank plus sparse matrix decomposition for accelerated dynamic mri with separation of background and dynamic components. Magn Reson Med.

[74] Gao H, Yu H, Osher S, Wang G (2011). Multi-energy ct based on a prior rank, intensity and sparsity model (prism). Inverse Probl.

[75] Cohen R, Zhang Y, Solomon O, Toberman D, Taieb L, van Sloun RJ, Eldar YC. Deep convolutional robust pca with application to ultrasound imaging. In: ICASSP 2019-2019 IEEE international conference on acoustics, speech and signal processing (ICASSP). new York: IEEE; 2019. p. 3212–6.

[76] Zhang H, Lin Z, Zhang C, Gao J (2015). Relations among some low-rank subspace recovery models. Neural Comput.

[77] Ding X, He L, Carin L (2011). Bayesian robust principal component analysis. IEEE Trans Image Process.

[78] Babacan SD, Luessi M, Molina R, Katsaggelos AK (2012). Sparse Bayesian methods for low-rank matrix estimation. IEEE Trans Signal Process.

[79] Zhao Q, Meng D, Xu Z, Zuo W, Zhang L. Robust principal component analysis with complex noise. In: International conference on machine learning. 2014. p. 55–63.

[80] Wright J, Ganesh A, Rao S, Peng Y, Ma Y. Robust principal component analysis: Exact recovery of corrupted low-rank matrices via convex optimization. In: Advances in neural information processing systems. 2009. p. 2080–8.

[81] Liu R, Lin Z, Wei S, Su Z. Solving principal component pursuit in linear time via $$l\_1$$ filtering. 2011. arXiv preprint arXiv:1108.5359.

[82] Mu Y, Dong J, Yuan X, Yan S. Accelerated low-rank visual recovery by random projection. In: CVPR 2011. New York: IEEE; 2011. p. 2609–16.

[83] Lin Z, Chen M, Ma Y. The augmented lagrange multiplier method for exact recovery of corrupted low-rank matrices. 2010. arXiv preprint arXiv:1009.5055.

[84] Anderson M, Ballard G, Demmel J, Keutzer K. Communication-avoiding qr decomposition for gpus. In: 2011 IEEE international parallel & distributed processing symposium. New York: IEEE; 2011. p. 48–58.

[85] Tang G, Nehorai A. Robust principal component analysis based on low-rank and block-sparse matrix decomposition. In: 2011 45th annual conference on information sciences and systems. New York: IEEE; 2011. p. 1–5.

[86] Klopp O, Lounici K, Tsybakov AB (2017). Robust matrix completion. Probab Theory Related Fields.

[87] Xu H, Caramanis C, Sanghavi S. Robust pca via outlier pursuit. In: Advances in neural information processing systems. 2010. p. 2496–504.

[88] Kim J, He Y, Park H (2014). Algorithms for nonnegative matrix and tensor factorizations: a unified view based on block coordinate descent framework. J Glob Optim.

[89] Lee DD, Seung HS (1999). Learning the parts of objects by non-negative matrix factorization. Nature.

[90] Van Benthem MH, Keenan MR (2004). Fast algorithm for the solution of large-scale non-negativity-constrained least squares problems. J Chemom J Chemom Soc.

[91] Javed S, Narayanamurthy P, Bouwmans T, Vaswani N. Robust pca and robust subspace tracking: a comparative evaluation. In: 2018 IEEE statistical signal processing workshop (SSP). New York: IEEE; 2018. p. 836–40.

[92] Lu C, Feng J, Chen Y, Liu W, Lin Z, Yan S. Tensor robust principal component analysis: exact recovery of corrupted low-rank tensors via convex optimization. In: Proceedings of the IEEE conference on computer vision and pattern recognition. 2016. p. 5249–57.

[93] Zhang Z, Ely G, Aeron S, Hao N, Kilmer M. Novel methods for multilinear data completion and de-noising based on tensor-svd. In: Proceedings of the IEEE conference on computer vision and pattern recognition. 2014. p. 3842–9.

[94] Goldfarb D, Qin Z (2014). Robust low-rank tensor recovery: models and algorithms. SIAM J Matrix Anal Appl.

[95] Li Yin, Yan Junchi, Zhou Yue, Yang Jie (2010). Optimum Subspace Learning and Error Correction for Tensors. Computer Vision – ECCV 2010.

[96] Kolda TG, Bader BW (2009). Tensor decompositions and applications. SIAM Rev.

[97] De Lathauwer L, De Moor B, Vandewalle J (2000). A multilinear singular value decomposition. SIAM J Matrix Anal Appl.

[98] De Lathauwer L, De Moor B, Vandewalle J (2000). On the best rank-1 and rank-(r 1, r 2,..., rn) approximation of higher-order tensors. SIAM J Matrix Anal Appl.

[99] Bayat M, Alizad A, Fatemi M. Multi-rate higher order singular value decomposition for enhanced non-contrast ultrasound Doppler imaging of slow flow. In: 2018 IEEE 15th international symposium on biomedical imaging (ISBI 2018). New York: IEEE; 2018. p. 1178–81.

[100] Zhang H, Cai J-F, Cheng L, Zhu J. Strongly convex programming for exact matrix completion and robust principal component analysis. 2011. arXiv preprint arXiv:1112.3946.

[101] Chen Z. Multidimensional signal processing for sparse and low-rank problems. PhD thesis, Citeseer. 2014.

[102] Cai J-F, Candès EJ, Shen Z (2010). A singular value thresholding algorithm for matrix completion. SIAM J Optim.

[103] Lin Z, Ganesh A, Wright J, Wu L, Chen M, Ma Y. Fast convex optimization algorithms for exact recovery of a corrupted low-rank matrix. Coordinated Science Laboratory Report no. UILU-ENG-09-2214, DC-246. 2009.

[104] Errico C, Pierre J, Pezet S, Desailly Y, Lenkei Z, Couture O, Tanter M (2015). Ultrafast ultrasound localization microscopy for deep super-resolution vascular imaging. Nature.

[105] Chee AJ, Alfred C (2017). Receiver-operating characteristic analysis of eigen-based clutter filters for ultrasound color flow imaging. IEEE Trans Ultrason Ferroelectr Freq Control.

[106] Urban A, Dussaux C, Martel G, Brunner C, Mace E, Montaldo G (2015). Real-time imaging of brain activity in freely moving rats using functional ultrasound. Nat Methods.

[107] Errico C, Osmanski B-F, Pezet S, Couture O, Lenkei Z, Tanter M (2016). Transcranial functional ultrasound imaging of the brain using microbubble-enhanced ultrasensitive Doppler. NeuroImage.

[108] Christensen-Jeffries K, Browning RJ, Tang M-X, Dunsby C, Eckersley RJ (2014). In vivo acoustic super-resolution and super-resolved velocity mapping using microbubbles. IEEE Trans Med imaging.

[109] Hintermüller M, Wu T (2015). Robust principal component pursuit via inexact alternating minimization on matrix manifolds. J Math Imaging Vis.

[110] Jensen JA. Field: A program for simulating ultrasound systems. In: 10th nordicbaltic conference on biomedical imaging, vol. 4, supplement 1, part 1. Citeseer; p. 351–3. 1996.

[111] Jensen JA, Svendsen NB (1992). Calculation of pressure fields from arbitrarily shaped, apodized, and excited ultrasound transducers. IEEE Trans Ultrason Ferroelectr Freq Control.

[112] Ashikuzzaman M, Gauthier CJ, Rivaz H (2019). Global ultrasound elastography in spatial and temporal domains. IEEE Trans Ultrason Ferroelectr Freq Control.

[113] Freund RM, Grigas P, Mazumder R (2017). An extended Frank-Wolfe method with “in-face” directions, and its application to low-rank matrix completion. SIAM J Optim.

[114] Nayak R, Fatemi M, Alizad A (2019). Adaptive background noise bias suppression in contrast-free ultrasound microvascular imaging. Phys Med Biol.

