# Supplementary material for: Clutter suppression in ultrasound: performance evaluation and review of low-rank and sparse matrix decomposition methods
Source: Biomed Eng Online. 2020 May 28;19:37. doi: 10.1186/s12938-020-00778-z (PMC7254711; doi:10.1186/s12938-020-00778-z)
Supplement: Supplementary file 1 — Additional file 1: Table S1. Information and abbreviations of DLSM algorithms. [file 12938_2020_778_MOESM1_ESM.pdf]

## Information and Abbreviations of DLSM Algorithms

| | Group | Abbreviation | Algorithm name | Authors |
|---|---|---|---|---|
| 1 | RPCA | RPCA | Robust Principal Component Analysis | De la Torre and Black [1] |
| 2 | RPCA | PCP | Principal Component Pursuit | Candes et al. [2] |
| 3 | RPCA | FPCP | Fast PCP | Rodriguez and Wohlberg [3] |
| 4 | RPCA | R2PCP | Riemannian Robust Principal Component Pursuit | Hintermüller and Wu [4] |
| 5 | RPCA | AS-RPCA | Active Subspace: Towards Scalable Low-Rank Learning | Liu and Yan [5] |
| 6 | RPCA | ALM | Augmented Lagrange Multiplier | Tang and Nehorai [6] |
| 7 | RPCA | EALM | Exact ALM | Lin et al. [7] |
| 8 | RPCA | IALM | Inexact ALM | Lin et al. [7] |
| 9 | RPCA | IALM-LMSVDS | IALM with LMSVDS | Liu et al. [8] |
| 10 | RPCA | IALM-BLWS | IALM with BLWS | Lin and Wei [9] |
| 11 | RPCA | APG-PARTIAL | Partial Accelerated Proximal Gradient | Lin et al. [7] |
| 12 | RPCA | APG | Accelerated Proximal Gradient | Lin et al. [7] |
| 13 | RPCA | DUAL | Dual RPCA | Lin et al. [7] |
| 14 | RPCA | SVT | Singular Value Thresholding | Cai et al. [10] |
| 15 | RPCA | ADM | Alternating Direction Method | Yuan and Yang [11] |
| 16 | RPCA | LSADM | LSADM | Goldfarb et al. [12] |
| 17 | RPCA | L1F | L1 Filtering | Liu et al. [13] |
| 18 | RPCA | DECOLOR | Contiguous Outliers in the Low-Rank Representation | Zhou et al. [14] |
| 19 | RPCA | RegL1-ALM | Low-Rank Matrix Approximation under Robust L1-Norm | Zheng et al. [15] |
| 20 | RPCA | GA | Grassmann Average | Hauberg et al. [16] |
| 21 | RPCA | GM | Grassmann Median | Hauberg et al. [16] |
| 22 | RPCA | TGA | Trimmed Grassmann Average | Hauberg et al. [16] |
| 23 | RPCA | STOC-RPCA | Online Robust PCA via Stochastic Optimization | Feng et al. [17] |
| 24 | RPCA | MoG-RPCA | Mixture of Gaussians RPCA | Zhao et al. [18] |
| 25 | RPCA | noncvxRPCA | Robust PCA via Nonconvex Rank Approximation | Kang et al. [19] |
| 26 | RPCA | NSA1 | Non-Smooth Augmented Lagrangian v1 | Aybat et al. [20] |
| 27 | RPCA | NSA2 | Non-Smooth Augmented Lagrangian v2 | Aybat et al. [20] |
| 28 | RPCA | PSPG | Partially Smooth Proximal Gradient | Aybat et al. [21] |
| 29 | RPCA | flip-SPCP-sum-SPG | Flip-Flop version of Stable PCP-sum solved by Spectral Projected Gradient | Aravkin et al. [22] |
| 30 | RPCA | flip-SPCP-max-QN | Flip-Flop version of Stable PCP-max solved by Quasi-Newton | Aravkin et al. [22] |
| 31 | RPCA | Lag-SPCP-SPG | Lagrangian SPCP solved by Spectral Projected Gradient | Aravkin et al. [22] |
| 32 | RPCA | Lag-SPCP-QN | Lagrangian SPCP solved by Quasi-Newton | Aravkin et al. [22] |
| 33 | RPCA | FW-T | SPCP solved by Frank-Wolfe method | Mu et al. [23] |
| 34 | RPCA | BRPCA-MD | Bayesian Robust PCA with Markov Dependency | Ding et al. [24] |
| 35 | RPCA | BRPCA-MD-NSS | BRPCA-MD with Non-Stationary Noise | Ding et al. [24] |
| 36 | RPCA | VBRPCA | Variational Bayesian RPCA | Babacan et al. [25] |
| 37 | RPCA | PRMF | Probabilistic Robust Matrix Factorization | Wang et al. [26] |
| 38 | RPCA | OPRMF | Online PRMF | Wang et al. [26] |
| 39 | RPCA | MBRMF | Markov BRMF | Wang and Yeung [27] |
| 40 | RPCA | TFOCS-EC | TFOCS with equality constraints | Becker et al. [28] |
| 41 | RPCA | TFOCS-IC | TFOCS with inequality constraints | Becker et al. [28] |
| 42 | RPCA | GoDec | Go Decomposition | Zhou and Tao [29] |
| 43 | RPCA | SSGoDec | Semi-Soft GoDec | Zhou and Tao [29] |
| 44 | RPCA | GreGoDec | Greedy Semi-Soft GoDec Algorithm | Zhou and Tao [30] |
| 45 | ST | GRASTA | Grassmannian Robust Adaptive Subspace Tracking Algorithm | He et al. [31] |
| 46 | ST | GOSUS | Grassmannian Online Subspace Updates with Structured-sparsity | Xu et al. [32] |
| 47 | ST | pROST | Robust PCA and subspace tracking from incomplete observations using L0-surrogates | Hage and Kleinsteuber [33] |
| 48 | ST | ReProCS | Provable Dynamic Robust PCA or Robust Subspace Tracking | Narayanamurthy and Vaswani [34] |
| 49 | ST | MEDRoP | Memory Efficient Dynamic Robust PCA | Narayanamurthy and Vaswani [35] |
| 50 | MC | PG-RMC | Nearly Optimal Robust matrix Completion | Cherapanamjeri et al. [36] |
| 51 | MC | FPC | Fixed point and Bregman iterative methods for matrix rank minimization | Ma et al. [37] |
| 52 | MC | GROUSE | Grassmannian Rank-One Update Subspace Estimation | Balzano et al. [38] |
| 53 | MC | IALM-MC | Inexact ALM for Matrix Completion | Lin et al. [7] |
| 54 | MC | LMaFit | Low-Rank Matrix Fitting | Wen et al. [39] |
| 55 | MC | LRGeomCG | Low-rank matrix completion by Riemannian optimization | Bart Vandereycken, 2013 [40] |
| 56 | MC | MC-logdet | Top-N Recommender System via Matrix Completion | Kang et al. [41] |
| 57 | MC | MC-NMF | Nonnegative Matrix Completion | Xu et al. [42] |
| 58 | MC | OP-RPCA | Robust PCA via Outlier Pursuit | Xu et al. [43] |
| 59 | MC | OptSpace | Matrix Completion from Noisy Entries | Keshavan et al. [44] |
| 60 | MC | OR1MP | Orthogonal rank-one matrix pursuit for low rank matrix completion | Wang et al. [45] |
| 61 | MC | RPCA-GD | Robust PCA via Gradient Descent | Yi et al. [46] |
| 62 | MC | ScGrassMC | Scaled Gradients on Grassmann Manifolds for Matrix Completion | Ngo and Saad [47] |
| 63 | MC | SVP | Guaranteed Rank Minimization via Singular Value Projection | Meka et al. [48] |
| 64 | MC | SVT | A singular value thresholding algorithm for matrix completion | Cai et al. [49] |
| 65 | LRR | EALM | Exact ALM | Lin et al. [7] |
| 66 | LRR | IALM | Inexact ALM | Lin et al. [7] |
| 67 | LRR | ADM | Alternating Direction Method | Lin et al. [50] |
| 68 | LRR | LADMAP | Linearized ADM with Adaptive Penalty | Lin et al. [50] |
| 69 | LRR | FastLADMAP | Fast LADMAP | Lin et al. [50] |
| 70 | LRR | ROSL | Robust Orthonormal Subspace Learning | Shu et al. [51] |
| 71 | TTD | 3WD | 3-Way-Decomposition | Oreifej et al. [52] |
| 72 | TTD | MAMR | Motion-Assisted Matrix Restoration | Ye et al. [53] |
| 73 | TTD | RMAMR | Robust Motion-Assisted Matrix Restoration | Ye et al. [53] |
| 74 | TTD | ADMM | Alternating Direction Method of Multipliers | Parikh and Boyd [54] |
| 75 | NMF | NMF-MU | NMF solved by Multiplicative Updates | unknown |
| 76 | NMF | NMF-PG | NMF solved by Projected Gradient | unknown |

| | | | | |
|---|---|---|---|---|
| 77 | NMF | NMF-ALS | NMF solved by Alternating Least Squares | unknown |
| 78 | NMF | NMF-ALS-OBS | NMF solved by Alternating Least Squares with Optimal Brain Surgeon | unknown |
| 79 | NMF | PNMF | Probabilistic Non-negative Matrix Factorization | unknown |
| 80 | NMF | ManhNMF | Manhattan NMF | Guan et al. [55] |
| 81 | NMF | NeNMF | NMF via Nesterov's Optimal Gradient Method | Guan et al. [55] |
| 82 | NMF | LNMF | Spatially Localized NMF | Li et al. [56] |
| 83 | NMF | ENMF | Exact NMF | Gillis and Glineur [57] |
| 84 | NMF | nmfLS2 | Non-negative Matrix Factorization with sparse matrix | Ji and Eisenstein [58] |
| 85 | NMF | Semi-NMF | Semi Non-negative Matrix Factorization | unknown |
| 86 | NMF | Deep-Semi-NMF | Deep Semi Non-negative Matrix Factorization | Trigeorgis et al. [59] |
| 87 | NMF | iNMF | Incremental Subspace Learning via NMF | Bucak and Gunsel [60] |
| 88 | NMF | DRMF | Direct Robust Matrix Factorization | Xiong et al. [61] |
| 89 | NTF | betaNTF | Simple beta-NTF implementation | Antoine Liutkus [62] |
| 90 | NTF | bcuNTD | Non-negative Tucker Decomposition by block-coordinate update | Xu and Yin [63] |
| 91 | NTF | bcuNCP | Non-negative CP Decomposition by block-coordinate update | Xu and Yin [63] |
| 92 | NTF | NTD-MU | Non-negative Tucker Decomposition solved by Multiplicative Updates | Zhou et al. [64] |
| 93 | NTF | NTD-APG | Non-negative Tucker Decomposition solved by Accelerated Proximal Gradient | Zhou et al. [64] |
| 94 | NTF | NTD-HALS | Non-negative Tucker Decomposition solved by Hierarchical ALS | Zhou et al. [64] |
| 95 | TD | HoSVD | Higher-order Singular Value Decomposition (Tucker Decomposition) | unknown |
| 96 | TD | HoRPCA-IALM | HoRPCA solved by IALM | Goldfarb and Qin [65] |
| 97 | TD | HoRPCA-S | HoRPCA with Singleton model solved by ADAL | Goldfarb and Qin [65] |
| 98 | TD | HoRPCA-S-NCX | HoRPCA with Singleton model solved by ADAL (non-convex) | Goldfarb and Qin [65] |
| 99 | TD | Tucker-ADAL | Tucker Decomposition solved by ADAL | Goldfarb and Qin [65] |
| 100 | TD | Tucker-ALS | Tucker Decomposition solved by ALS | unknown |
| 101 | TD | CP-ALS | PARAFAC/CP decomposition solved by ALS | unknown |
| 102 | TD | CP-APR | PARAFAC/CP decomposition solved by Alternating Poisson Regression | Chi et al. [66] |
| 103 | TD | CP2 | PARAFAC2 decomposition solved by ALS | Bro et al. [67] |
| 104 | TD | RSTD | Rank Sparsity Tensor Decomposition | Yin Li [68] |
| 105 | TD | t-SVD | Tensor SVD in Fourrier Domain | Zhang et al. 2013 [69] |
| 106 | TD | OSTD | Online Stochastic Tensor Decomposition | Sobral et al. [70] |

**Author details**

**References**


1. De la Torre, F., Black, M.J.: Robust principal component analysis for computer vision. In: Proceedings Eighth IEEE International Conference on Computer Vision. ICCV 2001, vol. 1, pp. 362–369 (2001). IEEE
2. Candès, E.J., Li, X., Ma, Y., Wright, J.: Robust principal component analysis? Journal of the ACM (JACM) **58**(3), 11 (2011)
3. Rodriguez, P., Wohlberg, B.: Fast principal component pursuit via alternating minimization. In: 2013 IEEE International Conference on Image Processing, pp. 69–73 (2013). IEEE
4. Hintermüller, M., Wu, T.: Robust principal component pursuit via inexact alternating minimization on matrix manifolds. Journal of Mathematical Imaging and Vision **51**(3), 361–377 (2015)
5. Liu, G., Yan, S.: Active subspace: Toward scalable low-rank learning. Neural computation **24**(12), 3371–3394 (2012)
6. Tang, G., Nehorai, A.: Robust principal component analysis based on low-rank and block-sparse matrix decomposition. In: 2011 45th Annual Conference on Information Sciences and Systems, pp. 1–5 (2011). IEEE
7. Lin, Z., Chen, M., Ma, Y.: The augmented lagrange multiplier method for exact recovery of corrupted low-rank matrices. arXiv preprint arXiv:1009.5055 (2010)
8. Liu, X., Wen, Z., Zhang, Y.: Limited memory block krylov subspace optimization for computing dominant singular value decompositions. SIAM Journal on Scientific Computing **35**(3), 1641–1668 (2013)
9. Lin, Z., Wei, S.: A block lanczos with warm start technique for accelerating nuclear norm minimization algorithms. arXiv preprint arXiv:1012.0365 (2010)
10. Cai, J., Candes, E., Shen, Z.: A singular value thresholding algorithm for matrix completion. arxiv, math. OC **810**, 1 (2008)
11. Yuan, X., Yang, J.: Sparse and low-rank matrix decomposition via alternating direction methods. preprint **12**, 2 (2009)
12. Goldfarb, D., Ma, S., Scheinberg, K.: Fast alternating linearization methods for minimizing the sum of two convex functions. Mathematical Programming **141**(1-2), 349–382 (2013)
13. Liu, R., Lin, Z., Wei, S., Su, Z.: Solving principal component pursuit in linear time via $l\_1$ filtering. arXiv preprint arXiv:1108.5359 (2011)
14. Zhou, X., Yang, C., Yu, W.: Moving object detection by detecting contiguous outliers in the low-rank representation. IEEE transactions on pattern analysis and machine intelligence **35**(3), 597–610 (2012)
15. Zheng, Y., Liu, G., Sugimoto, S., Yan, S., Okutomi, M.: Practical low-rank matrix approximation under robust l 1-norm. In: 2012 IEEE Conference on Computer Vision and Pattern Recognition, pp. 1410–1417 (2012). IEEE
16. Hauberg, S., Feragen, A., Black, M.J.: Grassmann averages for scalable robust pca. In: Proceedings of the IEEE Conference on Computer Vision and Pattern Recognition, pp. 3810–3817 (2014)
17. Feng, J., Xu, H., Yan, S.: Online robust pca via stochastic optimization. In: Advances in Neural Information Processing Systems, pp. 404–412 (2013)
18. Zhao, Q., Meng, D., Xu, Z., Zuo, W., Zhang, L.: Robust principal component analysis with complex noise. In: International Conference on Machine Learning, pp. 55–63 (2014)
19. Kang, Z., Peng, C., Cheng, Q.: Robust pca via nonconvex rank approximation. In: 2015 IEEE International Conference on Data Mining, pp. 211–220 (2015). IEEE
20. Aybat, N.S., Goldfarb, D., Iyengar, G.: Fast first-order methods for stable principal component pursuit. arXiv preprint arXiv:1105.2126 (2011)
21. Aybat, N.S., Goldfarb, D., Ma, S.: Efficient algorithms for robust and stable principal component pursuit problems. Computational Optimization and Applications **58**(1), 1–29 (2014)
22. Aravkin, A., Becker, S., Cevher, V., Olsen, P.: A variational approach to stable principal component pursuit. arXiv preprint arXiv:1406.1089 (2014)
23. Mu, C., Zhang, Y., Wright, J., Goldfarb, D.: Scalable robust matrix recovery: Frank–wolfe meets proximal methods. SIAM Journal on Scientific Computing **38**(5), 3291–3317 (2016)
24. Ding, X., He, L., Carin, L.: Bayesian robust principal component analysis. IEEE Transactions on Image Processing **20**(12), 3419–3430 (2011)
25. Babacan, S.D., Luessi, M., Molina, R., Katsaggelos, A.K.: Sparse bayesian methods for low-rank matrix estimation. IEEE Transactions on Signal Processing **60**(8), 3964–3977 (2012)
26. Wang, N., Yao, T., Wang, J., Yeung, D.-Y.: A probabilistic approach to robust matrix factorization. In: European Conference on Computer Vision, pp. 126–139 (2012). Springer
27. Wang, N., Yeung, D.-Y.: Bayesian robust matrix factorization for image and video processing. In: Proceedings of the IEEE International Conference on Computer Vision, pp. 1785–1792 (2013)
28. Becker, S.R., Candès, E.J., Grant, M.C.: Templates for convex cone problems with applications to sparse signal recovery. Mathematical programming computation **3**(3), 165 (2011)
29. Zhou, T., Tao, D.: Godec: Randomized low-rank & sparse matrix decomposition in noisy case. In: Proceedings of the 28th International Conference on Machine Learning, ICML 2011 (2011)
30. Zhou, T., Tao, D.: Greedy bilateral sketch, completion & smoothing. Journal of Machine Learning Research (2013)
31. He, J., Balzano, L., Lui, J.: Online robust subspace tracking from partial information. arXiv preprint arXiv:1109.3827 (2011)

32. Xu, J., Ithapu, V.K., Mukherjee, L., Rehg, J.M., Singh, V.: Gosus: Grassmannian online subspace updates with structured-sparsity. In: Proceedings of the IEEE International Conference on Computer Vision, pp. 3376–3383 (2013)
33. Hage, C., Kleinsteuber, M.: Robust pca and subspace tracking from incomplete observations using $\ell_0$-surrogates. Computational Statistics **29**(3-4), 467–487 (2014)
34. Narayanamurthy, P., Vaswani, N.: Provable dynamic robust pca or robust subspace tracking. IEEE Transactions on Information Theory **65**(3), 1547–1577 (2018)
35. Narayanamurthy, P., Vaswani, N.: Nearly optimal robust subspace tracking. arXiv preprint arXiv:1712.06061 (2017)
36. Cherapanamjeri, Y., Gupta, K., Jain, P.: Nearly optimal robust matrix completion. In: Proceedings of the 34th International Conference on Machine Learning-Volume 70, pp. 797–805 (2017). JMLR. org
37. Ma, S., Goldfarb, D., Chen, L.: Fixed point and bregman iterative methods for matrix rank minimization. Mathematical Programming **128**(1-2), 321–353 (2011)
38. Balzano, L., Nowak, R., Recht, B.: Online identification and tracking of subspaces from highly incomplete information. In: 2010 48th Annual Allerton Conference on Communication, Control, and Computing (Allerton), pp. 704–711 (2010). IEEE
39. Wen, Z., Yin, W., Zhang, Y.: Solving a low-rank factorization model for matrix completion by a nonlinear successive over-relaxation algorithm. Mathematical Programming Computation **4**(4), 333–361 (2012)
40. Vandereycken, B.: Low-rank matrix completion by riemannian optimization. SIAM Journal on Optimization **23**(2), 1214–1236 (2013)
41. Kang, Z., Peng, C., Cheng, Q.: Top-n recommender system via matrix completion. In: Thirtieth AAAI Conference on Artificial Intelligence (2016)
42. Xu, Y., Yin, W., Wen, Z., Zhang, Y.: An alternating direction algorithm for matrix completion with nonnegative factors. Frontiers of Mathematics in China **7**(2), 365–384 (2012)
43. Xu, H., Caramanis, C., Sanghavi, S.: Robust pca via outlier pursuit. In: Advances in Neural Information Processing Systems, pp. 2496–2504 (2010)
44. Keshavan, R.H., Montanari, A., Oh, S.: Matrix completion from noisy entries. Journal of Machine Learning Research **11**(Jul), 2057–2078 (2010)
45. Wang, Z., Lai, M.-J., Lu, Z., Fan, W., Davulcu, H., Ye, J.: Orthogonal rank-one matrix pursuit for low rank matrix completion. SIAM Journal on Scientific Computing **37**(1), 488–514 (2015)
46. Yi, X., Park, D., Chen, Y., Caramanis, C.: Fast algorithms for robust pca via gradient descent. In: Advances in Neural Information Processing Systems, pp. 4152–4160 (2016)
47. Ngo, T., Saad, Y.: Scaled gradients on grassmann manifolds for matrix completion. In: Advances in Neural Information Processing Systems, pp. 1412–1420 (2012)
48. Jain, P., Meka, R., Dhillon, I.S.: Guaranteed rank minimization via singular value projection. In: Advances in Neural Information Processing Systems, pp. 937–945 (2010)
49. Cai, J.-F., Candès, E.J., Shen, Z.: A singular value thresholding algorithm for matrix completion. SIAM Journal on Optimization **20**(4), 1956–1982 (2010)
50. Lin, Z., Liu, R., Su, Z.: Linearized alternating direction method with adaptive penalty for low-rank representation. In: Advances in Neural Information Processing Systems, pp. 612–620 (2011)
51. Shu, X., Porikli, F., Ahuja, N.: Robust orthonormal subspace learning: Efficient recovery of corrupted low-rank matrices. In: Proceedings of the IEEE Conference on Computer Vision and Pattern Recognition, pp. 3874–3881 (2014)
52. Oreifej, O., Li, X., Shah, M.: Simultaneous video stabilization and moving object detection in turbulence. IEEE transactions on pattern analysis and machine intelligence **35**(2), 450–462 (2012)
53. Ye, X., Yang, J., Sun, X., Li, K., Hou, C., Wang, Y.: Foreground–background separation from video clips via motion-assisted matrix restoration. IEEE Transactions on Circuits and Systems for Video Technology **25**(11), 1721–1734 (2015)
54. Parikh, N., Boyd, S., *et al.*: Proximal algorithms. Foundations and Trends® in Optimization **1**(3), 127–239 (2014)
55. Guan, N., Tao, D., Luo, Z., Shawe-Taylor, J.: Mahnmf: Manhattan non-negative matrix factorization. arXiv preprint arXiv:1207.3438 (2012)
56. Li, S.Z., Hou, X., Zhang, H., Cheng, Q.: Learning spatially localized, parts-based representation. CVPR (1) **207**, 212 (2001)
57. Gillis, N., Glineur, F.: On the geometric interpretation of the nonnegative rank. Linear Algebra and its Applications **437**(11), 2685–2712 (2012)
58. Ji, Y., Eisenstein, J.: Discriminative improvements to distributional sentence similarity. In: Proceedings of the 2013 Conference on Empirical Methods in Natural Language Processing, pp. 891–896 (2013)
59. Trigeorgis, G., Bousmalis, K., Zafeiriou, S., Schuller, B.: A deep semi-nmf model for learning hidden representations. In: International Conference on Machine Learning, pp. 1692–1700 (2014)
60. Bucak, S.S., Gunsel, B., Gursoy, O.: Incremental nonnegative matrix factorization for background modeling in surveillance video. In: 2007 IEEE 15th Signal Processing and Communications Applications, pp. 1–4 (2007). IEEE
61. Xiong, L., Chen, X., Schneider, J.: Direct robust matrix factorizatoin for anomaly detection. In: 2011 IEEE 11th International Conference on Data Mining, pp. 844–853 (2011). IEEE
62. Liutkus, A.: Simple to Use NMF/NTF with Beta Divergence. https://www.mathworks.com/matlabcentral/fileexchange/38109-simple-to-use-nmf-ntf-with-beta-divergence
63. Xu, Y., Yin, W.: A block coordinate descent method for regularized multiconvex optimization with applications to nonnegative tensor factorization and completion. SIAM Journal on imaging sciences **6**(3), 1758–1789 (2013)
64. Zhou, G., Cichocki, A., Xie, S.: Fast nonnegative matrix/tensor factorization based on low-rank approximation. IEEE Transactions on Signal Processing **60**(6), 2928–2940 (2012)
65. Goldfarb, D., Qin, Z.: Robust low-rank tensor recovery: Models and algorithms. SIAM Journal on Matrix Analysis and Applications **35**(1), 225–253 (2014)
66. Chi, E.C., Kolda, T.G.: On tensors, sparsity, and nonnegative factorizations. SIAM Journal on Matrix Analysis and Applications **33**(4), 1272–1299 (2012)
67. Kiers, H.A., Ten Berge, J.M., Bro, R.: Parafac2—part i. a direct fitting algorithm for the parafac2 model. Journal of Chemometrics: A Journal of the Chemometrics Society **13**(3-4), 275–294 (1999)
68. Li, Y., Yan, J., Zhou, Y., Yang, J.: Optimum subspace learning and error correction for tensors. In: European Conference on Computer Vision, pp. 790–803 (2010). Springer
69. Zhang, Z., Ely, G., Aeron, S., Hao, N., Kilmer, M.: Novel factorization strategies for higher order tensors: Implications for compression and recovery of multi-linear data. arXiv preprint arXiv:1307.0805 (2013)
70. Sobral, A., Javed, S., Ki Jung, S., Bouwmans, T., Zahzah, E.-h.: Online stochastic tensor decomposition for background subtraction in multispectral video sequences. In: Proceedings of the IEEE International Conference on Computer Vision Workshops, pp. 106–113 (2015)
